# Rings, Hexagons, Petals, and Dipolar Moment Sink-Sources: The Fanciful Behavior of Water around Cyclodextrin Complexes

**DOI:** 10.3390/biom10030431

**Published:** 2020-03-10

**Authors:** Pablo F. Garrido, Martín Calvelo, Rebeca Garcia-Fandiño, Ángel Piñeiro

**Affiliations:** 1 Departamento de Física de Aplicada, Facultade de Física, Universidade de Santiago de Compostela, E-15782 Santiago de Compostela, Spain; pablo.fernandez@usc.es; 2 Departamento de Química Orgánica, Center for Research in Biological Chemistry and Molecular Materials, Universidade de Santiago de Compostela, Campus Vida s/n, E-15782 Santiago de Compostela, Spain; martin.calvelo.souto@usc.es

**Keywords:** cyclodextrins, molecular dynamics simulations, bulk, interface, solvent order, sampling improvement

## Abstract

The basket-like geometry of cyclodextrins (CDs), with a cavity able to host hydrophobic groups, makes these molecules well suited for a large number of fundamental and industrial applications. Most of the established CD-based applications rely on trial and error studies, often ignoring key information at the atomic level that could be employed to design new products and to optimize their use. Computational simulations are well suited to fill this gap, especially in the case of CD systems due to their low number of degrees of freedom compared with typical macromolecular systems. Thus, the design and validation of solid and efficient methods to simulate and analyze CD-based systems is key to contribute to this field. The behavior of supramolecular complexes critically depends on the media where they are embedded, so the detailed characterization of the solvent is required to fully understand these systems. In the present work, we use the inclusion complex formed by two α-CDs and one sodium dodecyl sulfate molecule to test eight different parameterizations of the GROMOS and AMBER force fields, including several methods aimed to increase the conformational sampling in computational molecular dynamics simulation trajectories. The system proved to be extremely sensitive to the employed force field, as well as to the presence of a water/air interface. In agreement with previous experiments and in contrast to the results obtained with AMBER, the analysis of the simulations using GROMOS showed a quick adsorption of the complex to the interface as well as an extremely exotic behavior of the water molecules surrounding the structure both in the bulk aqueous solution and at the water surface. The chirality of the CD molecule seems to play an important role in this behavior. All together, these results are expected to be useful to better understand the behavior of CD-based supramolecular complexes such as adsorption or aggregation driving forces, as well as to introduce new methods able to speed up general MD simulations.

## 1. Introduction

The behavior of native cyclodextrins (CDs) in aqueous solution and at the air/water interface is much more complex than expected just considering their apparently simple molecular structure [1,2,3]. Consequently, the correct prediction of their properties and skills such as their solubility, their ability to adsorb at interfaces, or to encapsulate a variety of molecules is not straightforward; not to mention their propensity to aggregate forming different patterns in the bulk solution and at the water/air interface [4,5,6]. It is not a surprise then that the behavior of native and modified CDs, as well as that of the supramolecular complexes they form upon interacting with different types of molecules, is not trivial [7].

In spite of their great potential for a large number of applications, atomic level information of CD molecules, which is key to understand how structure relates to function, is really scarce. It is also important to remember that, like most functional supramolecular systems, CDs are not static and that only by visualizing them in action it is possible to understand how structure and function are connected. Moreover, a detailed structural-dynamic description at atomic resolution that explicitly includes the surrounding molecules is crucial to fully understand the behavior of supramolecular complexes since such behavior is strongly correlated with the entire multi-component system where they are embedded. Without the ability to understand dynamic structural changes, the design, development, and optimization of applications using CDs has been traditionally based on empirical trial-and-error essays as well as a fair degree of serendipity.

Computational simulations are commonly employed to study a large variety of systems including proteins, peptides, DNA, lipids, surfactants, and heterogeneous mixtures of different molecules in different solvents [8,9]. More specifically, molecular dynamics (MD) simulations are increasingly popular because they can provide structural, energetic, dynamic, and even mechanistic information. Computational studies of CDs are not so common, although a significant number of works based on MD simulations and docking of these molecules have already been published [10,11,12]. The number of degrees of freedom of single (native or modified) CDs is much lower than that of typical macromolecules. So, computational studies of these cyclic oligosaccharides are expected to be able to easily sample their conformational space and so to provide a good understanding of their behavior at the atomic level, as well as the connection of such behavior with macroscopic properties. Single CDs and CD complexes can be computationally studied in aqueous solution using relatively small simulation boxes and explicit solvent: ~3000 water molecules at room temperature and atmospheric pressure are typically enough to exhibit negligible direct interaction of these molecules with their periodic images. Using last-generation computational resources, it is feasible to get atomistic trajectories of such systems in the microsecond timescale. However, CDs and CD complexes at concentrations employed in typical applications often aggregate, adsorb to interfaces, penetrate other media such as lipid bilayers, and acquire new levels of organization both in bulk solution and at interfaces. The study of such aggregates requires longer timescales and larger simulation boxes, and so, much more computational effort than that required for the study of single molecules. The implementation of methods to increase the sampling of the conformational space at lower computational cost is then convenient to better approach the convergence of the most important properties of the studied systems. A practical general solution to speed up MD simulations is to use the so called coarse-grained (CG) force fields, where groups of atoms are represented by single beads [13], hence decreasing the number of degrees of freedom, mainly those that move faster. This also allows increasing the time step for the integration of the motion equations by more than one order of magnitude, as well as to smooth the energy landscape, avoiding kinetically trapped states and facilitating the evolution of the system towards the equilibrium. This method proved to work exceptionally well mainly for lipid membranes. Using this approach, it is more difficult to describe the behavior of other molecules -such as proteins or DNA- in a realistic way, so internal restraints are typically used to overcome obvious artifacts [14]. In any case, the atomistic structure of the systems simulated at CG resolution can be recovered by applying different algorithms [15,16]. CG parameterizations of CDs have been made in the past [17] but they are not expected to be fully reliable since the larger van der Waals radius of CG beads significantly reduces the volume of the cavity, thus seriously affecting their ability to encapsulate molecules as well as the accessible conformational space of the system. An alternative to save computational time is the use of multiple time-step algorithms such as RESPA (Reference System Propagator Algorithm) [18,19], but they have proved to introduce serious artifacts in some cases [20,21]. A different and very simple general method to efficiently increase the sampling of MD simulations was proposed by Feenstra et al. 20 years ago [22]. The method consists of transferring mass from heavy atoms to hydrogens (Hs) to slow their movement. This allows significantly increasing the time step for the integration of the motion equations. As a result, it is possible to reach longer trajectories as well as to smooth the energy landscape (as in CG simulations) by keeping the atomistic resolution of the system (in contrast to CG simulations). It is worth mentioning that the mass of the atoms is definitely much less critical for equilibrium properties than topological features (bond distances, angles, dihedrals, torsions, etc.), van der Waals, and electrostatic interactions. This can be experimentally demonstrated by the fact that structural and thermodynamic properties of many deuterated systems are indistinguishable from those of the equivalent hydrogenated structures (this has been specifically verified for CD systems) [23]. The Feenstra method, also called the HMR (hydrogen mass repartitioning) method, has been tested for many systems providing good results [24,25]. CD systems have also been simulated using this method for all-atom (AA) force fields with a mass transfer of 2 Da per H and a time step of 4 fs instead of the standard 2 fs [26]. In general, AA force fields allow transferring a maximum of 2 Da from heavy to the bound H atoms due to the possible presence of methyl groups. A mass transfer of 2 Da from a single C atom to three H implies reducing the mass of such carbon atom from 12 to 6 Da. This makes it impossible to transfer more mass without leaving the carbon lighter than the Hs to which it is bound. In contrast to AA force fields, the HMR method applied to united atom (UA) force fields such as GROMOS allows transferring up to 3 Da from heavy atoms to the bound polar H atoms (non-polar H atoms are implicit in this force field and so not explicit methyl groups nor Hs in aliphatic chains are present in this case). Consequently, using the mass transfer approach, the time step in UA force fields could be increased up to 7 fs, instead of 4 fs. It is worth to test the effect of a direct mass increase in the explicit Hs of UA force fields, avoiding the repartition proposed by Feenstra to increase the time step. The application of this method would not have the limitation implicit in AA force fields due to the large number of explicit H atoms present in that representation, which would imply a significant increment of mass, thus making the movement of the total molecule too slow. As a consequence, the increase of mass in AA force fields could reduce the sampling even if the time step is increased. The mass increase of just polar Hs from 1 to 2 Da is justified based on the experimental fact that only the polar H atoms are spontaneously deuterated (thus doubling their mass) when they are solvated in deuterated water [1,23]. In the present work, we propose to also test the mass increase of just the polar H up to 4 Da. This is expected to virtually mimic the behavior of the molecules with a heavier isotope of H (quadium) without the risk of subtracting too much mass to the bound carbon atoms, thus compromising the thermodynamic behavior of the global system. This last method will be named H2Q from here on. To our knowledge, the HMR method using UA force fields has not been tested yet for CD systems and the H2Q method has been marginally essayed (a literature revision is included in [22]) but not analyzed in detail.

**Figure 1:**
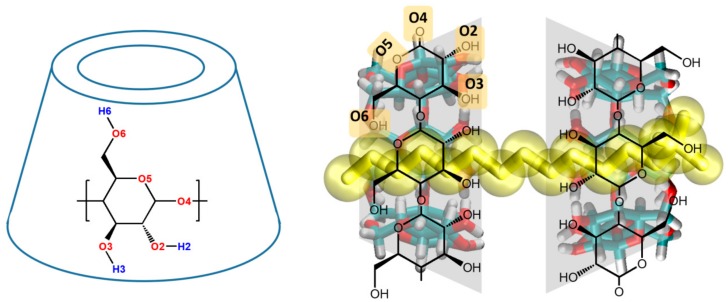
Two-dimensional representation of an α-cyclodextrin (CD) (left) and of an α-CD_2_SDS_1_ complex (right) with the oxygen atoms labelled. Just the polar H atoms are shown in the left figure. The surfactant molecule is represented in yellow sticks and transparent spheres while the two CDs in the complex are presented in lines and sticks.

The validation of any new methodology implies the comparison to a recognizable reference method to demonstrate equivalence of the obtained results. The selection of a system sensitive enough to subtle and precise discrepancies among the different methodologies is also crucial. In the present paper, we will focus on the behavior of the supramolecular 2:1 complex formed by α-CD and sodium dodecyl sulfate (SDS) (see Figure 1) in the bulk of aqueous solutions and also at water/air interfaces. CD-surfactant mixtures are especially interesting because they proved to exhibit interesting aggregation, mechanical, and adsorption properties under certain experimental conditions (concentration range and temperature) where the presence of 2:1 complexes dominate the solution [4,5,6,27,28,29,30] but the molecular interactions leading to such behavior are unclear. This can be illustrated by an specific example: The adsorption of α-CD_2_SDS_1_ complexes at interfaces leading to a monolayer has been conclusively demonstrated by different methods including surface tension, rheology, ellipsometry, Brewster angle microscopy (BAM), atomic force microscopy (AFM), and neutron reflectometry (NR), [5,6,23] although the adsorption driving force of those structures at the interface are still unknown. Additionally, preliminary work from our group indicates that this system is extremely sensitive to the parameterization, as will be shown in the present work. The selection of this highly sensitive system for which a lot of experimental information is available, and its study both in bulk and in the presence of an air/water interface, will allow testing several force field parameterizations as well as methods to speed up MD simulations of CD complexes at the time that the obtained information can be connected to the macroscopic properties of the system. The essayed methods could be later trustingly employed in larger and longer scale studies of similar systems. We will especially focus on the detailed analysis of the packing and structure of the solvent around the target complex, assuming that they have an important impact in its behavior, including their adsorption or penetration to media of different polarity, as well as to define their stoichiometry, topology, and also the stability of the corresponding structures. The role of the solvent structure for the aggregation and adsorption of different molecules has been discussed for many decades in the context of the hydrophobic effect [31]. However, the organization of the solvent in the immediate environment of solutes is not commonly characterized in detail and so the impact of such organization is often implicitly ignored. The strong correlation between the order of the lipids induced by pore proteins or modified carbon nanotubes in a membrane and the toxicity of the systems [32] has been recently reported. The structure of water around proteins has also been widely analyzed [33,34]. In the case of CD systems, many interaction mechanisms are unknown, and they are simply ignored in benefit of experimentally improving formulations just based on trial-and-error essays. The order of the solvent around CD complexes is expected to be key to explain many of such mechanisms, including how they aggregate, adsorb and organize at interfaces, or sequester molecules from lipid membranes. Several studies have analyzed this behavior for native and modified CDs using different methods [35,36,37] and some groups called the attention on the order of water around CD complexes [26], but no detailed studies have been performed on this latter issue.

In what follows, we will describe a number of methods employed to simulate CD-based complexes in the water solution and at water/air interfaces. The α-CD_2_SDS_1_ structure will be taken as a reference system for the present study. Then, the obtained results will be analyzed in detail with specific strength in the local density and order of water molecules around the complex using different simulation methods.

## 2. Materials and Methods

### 2.1. Set Up of the Simulation Boxes and MD Simulation Parameters

Several sets of MD simulations were performed starting from one preassembled α-CD_2_SDS_1_ complex located at random positions and orientations in pre-equilibrated 5 × 5 × 5 nm^3^ water boxes. The GROMOS [38] and AMBER [39,40] force fields were tested. For the simulations with GROMOS, the topology of SDS was built based on the 54a7 parameters and SPC water molecules were employed as explicit solvent [41]. For the α-CD molecule, different GROMOS parameterizations were essayed in order to slow down high-frequency motions thus allowing to increase the time step for the integration of the motion equation (see Section 2.2). TIP3P water molecules [42] were used for all the simulations with AMBER [39,40], and three different parameterizations were also employed for the SDS and α-CD molecules in this force field (see Section 2.2). The initial structure of the complex was taken from previous works [43]. For the studies at the interface, the Z dimension of the water boxes was enlarged by a factor of three thus getting 5 × 5 × 15 nm^3^ boxes with two water/air interfaces parallel to the XY plane. In all cases, one Na^+^ ion was introduced using the *genion* tool from the GROMACS 2018 package to neutralize the system. The same simulation parameters were employed for all the simulations, regardless the parameterization method. First, the simulation boxes were energy minimized using the steepest descent method. Approximately 500-ns-long MD trajectories for the simulations based on the GROMOS force field and 150-ns-long trajectories for the simulations using AMBER, all of them at 283 K, were generated. This temperature was employed because both the adsorption and the formation of 2:1 complexes are much more favorable at 283 K than at 298 K [5,6,23]. The NPT ensemble was employed for the simulations in solution and in the NVT ensemble for the simulation in the presence of water/air interfaces. Between one and three replicas of every simulation using different initial random velocities from a Maxwell–Boltzmann distribution at 283 K were performed. The GROMACS 2018 engine [44,45] was employed to get all the MD trajectories. The temperature was controlled using the V-rescale thermostat [46] with a coupling constant of 0.1 ps. For the simulations at constant pressure (1 bar), a Parrinello-Rahman barostat [47] with a compressibility of 4.5·10^−5^ bar^−1^ was employed. The coupling constant for the barostat was 0.5 ps for the simulations with a time step of 2 fs and 1.5 ps for the simulations with a time step of 7 fs. Long range electrostatic interactions were calculated using the particle mesh Ewald method [48,49] with a real-space cutoff of 1.2 nm, a 0.15 nm spaced grid, and fourth-order B-spline interpolation. The Ewald sum in three dimensions with a correction term (EW3DC) was used to avoid artifacts due to interactions between periodic images in the Z direction for the simulations with slab geometry. The equations of motion were integrated using the leapfrog method [50]. The SETTLE [51] algorithm was employed to constrain the bond lengths and angles of water molecules while the LINCS [52] algorithm was employed to constrain the bond lengths of the CD molecules. During the MD simulations, coordinates and energies were stored every 10 ps for analysis.

### 2.2. Parameterization Methods

Five types of simulations were performed using a standard parameterization of the GROMOS 54a7 force field for the SDS molecule and different modifications for α-CD molecule in order to increase the sampling (see Introduction section), namely: (i) Simulations using the GROMOS 54a7 force field [38] based on a parametrization of the glucopyranoside (GPU) units as building blocks [53] and using a time step of 2 fs for the integration of the motion equations (**G_2**); (ii) an alternative parameterization for the CDs based on G_2 but including the transference of 3 Da of mass to the H atoms from the bounded heavy atoms (O2, O3 or O6) and using the same time step (**HMR_2**); (iii) the latter parameterization (using the mass transfer to the H atoms) but with a time step of 7 fs (**HMR_7**) [22]; (iv) another alternative parameterization for the CDs also based on the G_2 parameterization but increasing the mass of the polar H atoms (H2, H3, and H6) from 1 to 4 Da, while the mass of the rest of the atoms remains unchanged, and using a time step of 2 fs (**H2Q_2**); and (v) the latter parameterization (mass increase in the polar H atoms of 3 additional Da) but using a time step of 7 fs (**H2Q_7**). Additionally, three different AMBER/GAFF parameterizations for the CD molecule were employed: (i) A first parameterization obtained by applying the *antechamber* [54,55] tool to the whole molecule and using RESP (Restrained Electrostatic Potential) charges (**AMBER RESP**); (ii) a second parameterization also using RESP charges and the antechamber tool applied just to the methylated GPU rings, which were then pasted together to form the topology of the whole CD (this method will be named **AMBER BB-RESP**); and (iii) a last parameterization equivalent to the previous one, i.e., using the GPUs as building blocks, but using AM1-BCC charges instead of RESP (this will be named **AMBER BB-AM1**). The SDS molecule was parameterized as a single entity in all cases, using the RESP and AM1-BCC charges, thus obtaining two different parameter sets. All the simulations using GROMOS were performed with the SPC water model [41] while the simulations with AMBER/GAFF were performed using the TIP3P water model [42,56] for the solvent. For the simulations using AMBER, no modification in the mass of the H atoms was performed.

### 2.3. Analysis of the Trajectories

For the simulations in the bulk solution, the 12 O4 atoms (Figure 1) of the two CDs were employed to fit the structure of the complex along each trajectory. Additionally, in order to facilitate the analysis, the segment joining the center of the O4 atoms of each CDs was aligned with one of the coordinate axes. The behavior of the water molecules around the complexes and the internal motion of the different CD atoms within the complex were analyzed in three different ways: (i) Water density maps for 3-Å-width slices perpendicular to the symmetry axis of the complex were determined using the last 10,000 frames of each trajectory with a resolution of 0.5 × 0.5 Å^2^. A total of 9 slices per structure were set, taking the center of the 12 O4 atoms of the two CDs as a reference. The first slice includes 1.5 Å towards each CD while the remaining 8 slices are located between 1–4, 4–7, 7–10, and 10–13 Å towards each CD of the complex. Thus, the segment going from 1 to 1.5 Å in the direction of each CD is accounted for in two different slices. In order to assess the specificity in the local orientation of the water molecules, the average dipolar moment was determined for the same slices, over the same number of frames, and for the same resolution. (ii) A single 3-Å-width slice parallel to and including the symmetry axis of the complex was taken and again the density of water molecules as well as their average dipolar moment in that plane was determined using the same resolution and frames from the MD trajectories; and (iii) 13 1-Å-width concentric shells centered in the symmetry axis of the complex were taken and the same analysis was performed using cylindrical coordinates.

**Figure 2:**
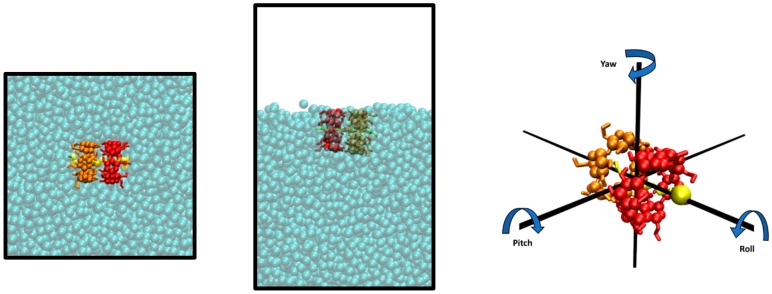
Representation of simulation boxes with an α-CD_2_SDS_1_ complex in the bulk aqueous solution (left) and in the presence of an air/water interface (middle). The supramolecular structure in the second image appears adsorbed with the symmetry axis parallel to the interface. Definition of the yaw, pitch, and roll angles for an α-CD_2_SDS_1_ complex (right). The three rotation angles are defined with respect to the interface shown in the central image. The CD molecules that are closer (CD1) and further (CD2) from the SDS polar head in the complex are represented in red and orange, respectively, with the glucopyranoside (GPU) rings in sphere representation and the hydroxyl groups in sticks. The sodium dodecyl sulfate (SDS) molecule is in yellow sticks, with the Sulphur atom as a sphere.

For the simulations of a single complex in the presence of water/air interfaces, the final conformation, regardless whether or not it is located at the water surface, was aligned with one of the coordinate axes and the resulting structure was employed to align the whole trajectory again using the O4 atoms as a reference. The distance between the center of the O4 atoms and the center of mass of the water, as well as the yaw, pitch, and roll angles, referred to the water surface, as a function of time were determined (see Figure 2). The hydration of each GPU unit within the complex, also as a function of time, was also determined using cutoff values of 3 and 5 Å around any atom of these rings. The same calculation was performed taking as a reference just the center of each ring with the same cutoff values measured from this point. The water density maps and dipolar moment analysis were determined for the trajectory segments where each of the six rings of one of the CDs exhibits the minimum hydration, thus guaranteeing that such a ring is orientated towards the air in when the structure is adsorbed. Thus, for the simulations at the interface, a set of density maps and dipolar moment distribution plots was obtained for each GPU group.

For all the simulations, the number of CD-CD and CD-water hydrogen bonds (H-bonds), as well as their lifetime, were also determined using the *hbonds* tool from the GROMACS 2018 package. The rest of the analysis was performed using our own code based on the *MDanalysis* python package [57,58].

## 3. Results

**Figure 3:**
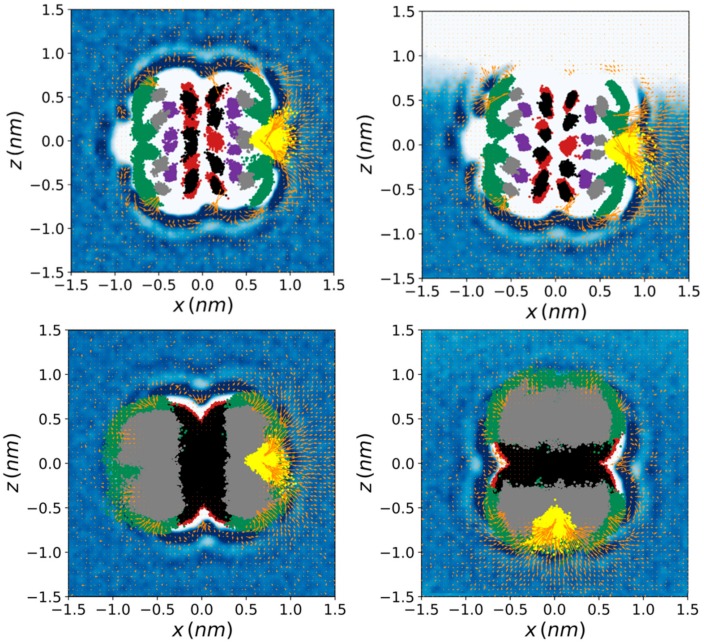
Water density (blue-white gradient from highest to lowest) and average water dipole moment (orange vectors) around the α-CD_2_SDS_1_ complex in the aqueous solution (left) and in the presence of water/air interface (right) for four trajectories generated using the GROMOS force field with the G_2 method (original GROMOS 54a7 force field with 2 fs time step) (top) and the AMBER/GAFF building block parameterization based on GPU rings with RESP charges (bottom). The O2 (red), O3 (black), O4 (purple), O5 (grey), and O6 (green) atoms of the two CDs comprising the complex, together with the sulfur atom of the SDS molecule (yellow), are also represented. The last 10,000 frames of one trajectory with the complex aligned to the O4 atoms were employed for these calculations. A resolution of 0.5 × 0.5 Å^2^ in the XZ plane for a 3 Å width slice perpendicular to the Y axis and centered in the symmetry axis of the complex was considered to generate the plot.

First, 500-ns-long trajectories of one α-CD_2_SDS_1_ complex in the bulk aqueous solution and exposed to the water/air interface were generated using five different simulation methods for the GROMOS force field: G_2, HMR_2, HMR_7, H2Q_2, and H2Q_7. For the simulations using the three different parameterizations of the AMBER/GAFF force field, 150-ns-long trajectories, also in the bulk solution and at the water/air interface, were generated. In short, the complexes were stable in all the MD simulations. For the simulations using GROMOS in the presence of interfaces, they were spontaneously adsorbed during the first few ns and remained floating during the rest of the trajectory with the symmetry axis parallel to the water surface (Figure 2 (middle) and Figure 3). The complexes were much less tight in the simulations using AMBER/GAFF and, in contrast with experimental evidences and with the results obtained with GROMOS, they did not adsorb to the interface using this force field. When they reached the interface by diffusion using AMBER/GAFF, they stayed with the symmetry axis perpendicular to the surface during a few ns and then they went back to the bulk solution. Thus, the results from the two force fields, regardless the parameterization method, were opposite in this sense. The next sections will present the results obtained from the G_2 method in the bulk aqueous solution, used as reference for the validation of the rest of the methodologies, and a description of the main similitudes and differences is performed.

### 3.1. Simulations in the Bulk Aqueous Solutions Using the G_2 Metho

**Figure 4:**
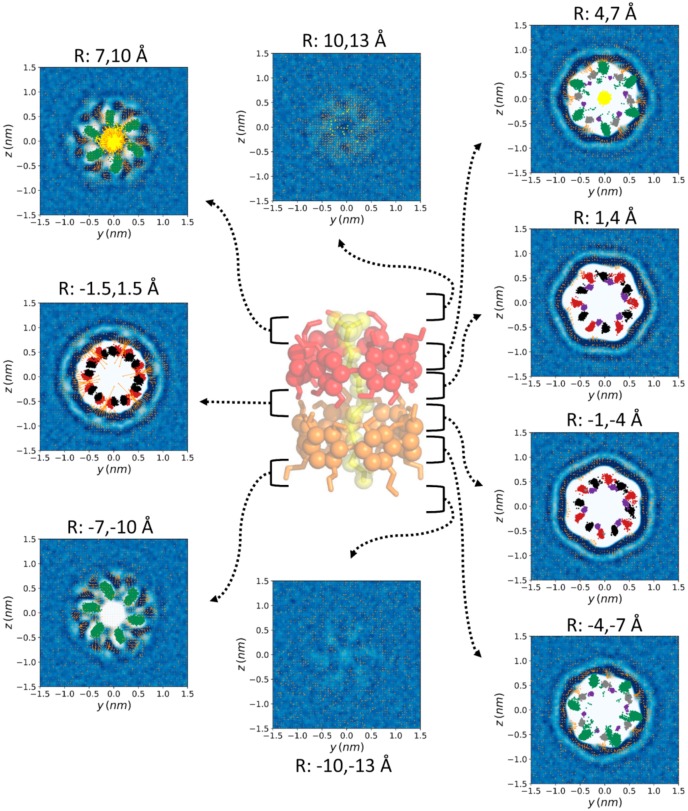
Water density (blue–white gradient from highest to lowest) and average water dipole moment (orange vectors) around the α-CD_2_SDS_1_ structure in aqueous solution for a trajectory generated using the G_2 method (original GROMOS 54a7 force field with 2 fs time step). Nine 3 Å width slices perpendicular to the symmetry axis of the complex and comprising the atoms at the indicated distances (R) from the center of the structure, were considered. The position of each slice is indicated by the brackets and arrows. The O2 (red), O3 (black), O4 (purple), O5 (grey), and O6 (green) atoms of the two CDs comprising the complex, together with the sulfur atom of the SDS molecule (yellow), are also represented. The last 10,000 frames of one trajectory with the complex aligned to the O4 atoms were employed for these calculations. A resolution of 0.5 × 0.5 Å^2^ in the YZ plane was considered to generate each plot. Equivalent plots for the rest of the studied parameterization methods are shown in the Supplementary Materials.

**Figure 5:**
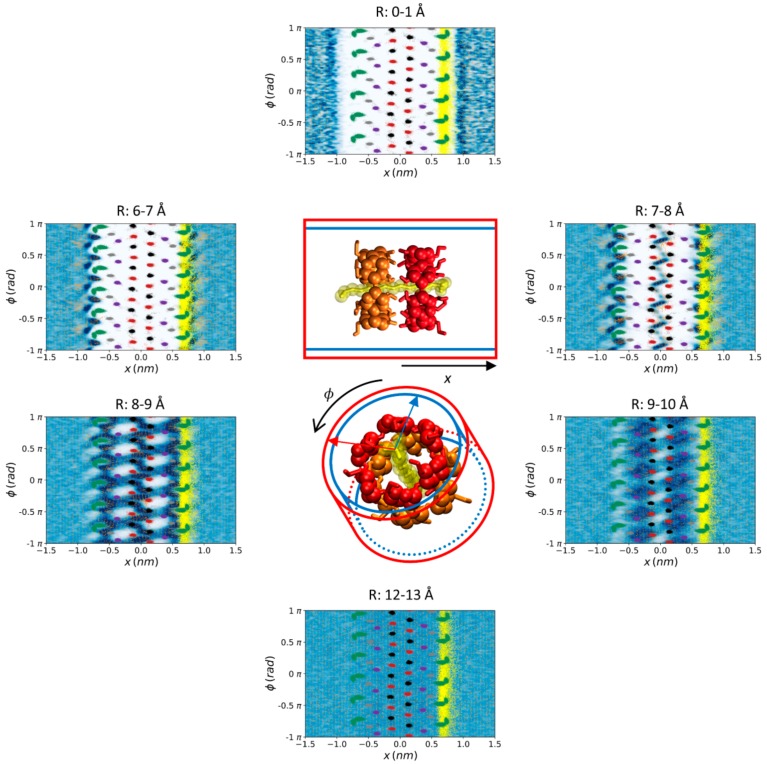
Water density (blue–white gradient from highest to lowest) and average water dipole moment (orange vectors) around the α-CD_2_SDS_1_ structure in aqueous solution for a trajectory generated using the G_2 method (original GROMOS 54a7 force field with 2 fs time step). Thirteen 1 Å thick coaxial shells centered in the symmetry axis of the complex were considered but only the more representative six are shown with the corresponding maximum and minimum radius indicated in each image. Cylindrical coordinates are employed, for clarity. The distances to the symmetry axis corresponding to each shell are indicated in the figure. The O2 (red), O3 (black), O4 (purple), O5 (grey), and O6 (green) atoms of the two CDs comprising the complex, together with the sulfur atom of the SDS molecule (yellow), are represented in all the images as a reference even though they are not within the indicated radii. The last 10,000 frames of one trajectory with the complex aligned to the O4 atoms were employed for these calculations. A resolution of 0.5 × 0.5 Å^2^ in the φx plane was considered to generate each plot. Equivalent plots for the rest of the studied parameterization methods are shown in the Supplementary Materials.

The water density map as well as the local dipole moment of the water molecules as a function of the position was determined in several slices (see Methods) perpendicular and parallel to the symmetry axis of the complex (Figure 3 and Figure 4), as well as in cylindrical coaxial shells centered in the same symmetry axis (Figure 5). The slice parallel to and centered in the nanocylinder symmetry axis showed a fanciful distribution and orientation of the water molecules around the 2:1 structure (Figure 3). The water had three 1.5-Å-width high–low–high-density layers, respectively, before reaching the bulk behavior. The water density distribution along the complex symmetry axis was very similar on both sides of the structure (i.e., for both CD molecules) but the water orientation changed significantly as a function of the distance to the oxygen atoms of the CDs (O2, O3, O4, O5, and O6) as well as of the distance to the sulfur atom of the SDS head. There were four zones where the water orientation was very specific, namely around the O6 atoms of both CDs, around the O2-O3 atoms of the CD that is closer to the SDS head, and a last region surrounding the sulfur atom of the SDS. The highly orientated water molecules were located in the first layer around the complex, where the density of the solvent was high. There were also zones where the water density was significantly higher compared to the bulk solution but where the average dipole moment of water molecules was negligible. The orientation of the water molecules in the second and third 1.5-Å-width layers around the supramolecular structure (with low and high density, respectively) also canceled the average dipolar moment. In addition to the water density and orientation, the position of the CD oxygen atoms throughout the last 10,000 frames of the studied trajectories was represented (Figure 3, in red, black, purple, grey, and green, for O2, O3, O4, O5, and O6, respectively). The motion amplitude of the different oxygen atoms was much larger for O6 than for O2, O3, O4, and O5; and slightly larger for O2 and O3 than for O4 and O5. This justifies using O4 as reference atoms to align CD structures.

The same analysis was performed for nine additional 3-Å-width slices along and perpendicular to the nanocylinder symmetry axis (Figure 4). This view of the water density and ordering allowed a more detailed perspective of the water around the complex. In contrast to the density profile, the water dipolar moment of equivalent slices (at the same distance from the center of the CD-CD interface) for both CDs was significantly different. The reason for this difference is clearly the proximity to the charged SDS head, as shown in the perpendicular slice (Figure 3). The petal-shaped density profiles observed for the slices between 7 and 10 Å from the center of the CD-CD interface revealed a highly anisotropic motion of the O6 atoms of both CDs (Figure 3, Figure 4 and Figure 5) likely due to the chirality of GPU rings of the CD molecules. It can be clearly seen that the orientation of the petals was clockwise and counterclockwise in the CDs that were further and closer to the head of the SDS molecule, respectively. This is because the O6 atoms producing this motion were orientated towards opposite directions along the symmetry axis of the complex. This particular motion of the O6 atoms seemed to be accompanied by water molecules since a high-density layer with similar geometry appears for both CDs. An interesting transition from a circular to hexagonal and again to circular density profile was observed for the slices centered at the interface between both CDs, as well as for those between 1–4 Å and 4–7 Å on both sides of the structure. This apparent shift results from the six-ring topology of both CDs combined with the opposite orientation of one CD with respect to the other in the complex. The GPU rings of both CD molecules were perfectly aligned, forming quite stable O2-H2-O3 and O2-H3-O3 intermolecular CD1-CD2 H-bonds (Table 1). The hexagons formed by the O5 atoms of both CDs were rotated 60 degrees with respect to each other in this conformation, which fit exactly with the relative orientation of the two hexagons appearing for the two slices at 1–4 Å from the center of the complex. Notably, there were six specific sources of the dipole moment vector field in the slice corresponding to the interface between both CDs. Such dipole moments had radial symmetry in this plane and the location of the corresponding sources was slightly shifted towards the head of the SDS molecule, as it had been previously mentioned (Figure 3 and Figure 4). This was reflected in significant differences between both CDs in the H-bond pattern and lifetimes involving O2-H2 and O3-H3 hydroxyls (see below). The O5 atoms were in the slices at 4–7 Å from the center of the complex while the O4 and O6 atoms of the CDs, as well as the sulfur atom of the surfactant molecule, fluctuated between the same slice and that at 7–10 Å. As in the slices corresponding to ±1.5 Å and 1–4 Å, the three high–low–high-density water layers between the solute and the bulk solution were perfectly visible at 4–7 Å. In this case, the hexagonal symmetry was lost, probably due to the influence of the O6 atoms movement. For the CD that was closer to the SDS head (CD1), some of these water molecules were significantly ordered in this slice. Finally, for the most external layers corresponding to 10–13 Å from the center of the structure, only the effect of the solute in the water molecules, translated into a petal-shaped density profile, can be seen. Some order in the water molecules that were closer to the SDS head can also be envisaged from the orientation of their dipole moments in this region.

The analysis shown in Figure 5 is complementary to those of Figure 3 and Figure 4. Thirteen 1-Å-thick coaxial shells centered in the symmetry axis of the complex were considered and cylindrical coordinates were employed. It can be noticed that the deepest water molecules appeared at 7–8 Å from the center of the structure, just between the O2 and O3 atoms, i.e., at the interface between both CDs. At 8–9 Å, the shape of the rings can be perfectly appreciated from the high density of water around them. The complementary profile was observed between 9–10 Å while the water density was uniform at more than 12 Å from the center of the complex. Overall, these complementary views of the water density, oxygen atoms, and water dipolar moment indicate that the solvent fits perfectly well to the complex structure.

**Figure 6:**
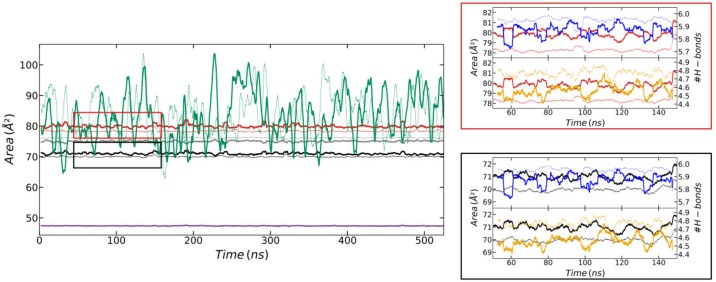
Area of the hexagons formed by the six O2 (red), O3 (black), O4 (purple), O5 (grey), and O6 (green) atoms of the CD molecules that are closer (solid thick lines) and further (dotted thin lines) from the SDS head. The right-upper plot (red-squared) shows the area of the hexagons formed by the six O2 of CD1 (thick red line) and CD2 (thin red line) between 50 and 150 ns together with: (i) The intramolecular H-bonds between the hydroxyl groups 2 and 3 of the CD that is closer and further to the SDS head (thick and thin blue lines, respectively); and (ii) the intermolecular H-bonds between the hydroxyl groups 2 and 3 of the first CD and second CD (thick yellow line) and vice versa (thin yellow line). The right-lower plot (black-squared) shows the same H-bond information as in the red-squared box, compared to the area of the hexagon formed by the six O3 of CD1 (thick black line) and CD2 (thin black line). This figure was obtained from the simulation of the α-CD_2_SDS_1_ structure in aqueous solution for a trajectory generated using the G_2 method (original GROMOS 54a7 force field with 2 fs time step). All the curves were moving-averaged over 3.5 ns in order to remove noise. Equivalent plots for the rest of the studied parameterization methods are shown in the Supplementary Materials.

In order to study the internal motion of the CD molecules, the area of the hexagons formed by the different oxygen atoms of the two CDs was monitored as a function of time (see Figure 6). The significant difference between the areas of the hexagons formed by O2 and those formed by O3 can be explained by the intramolecular and intermolecular H-bond pattern involving the corresponding hydroxyl groups. The hydroxyls containing O2 always donate the H2 atom to the O3 atom of the GPU unit in the opposite CD, thus participating in an intermolecular H-bond. In turn, the hydroxyls containing the O3 atoms always donate the corresponding H3 to the O2 atom belonging to the contiguous GPU group within the same CD, thus participating in an intramolecular H-bond. Experimental evidence of this specific behavior has been previously observed [59]. The correlation between the areas of the hexagons formed by the different oxygen atoms and the H-bonds of the corresponding hydroxyl groups is illustrated in Figure 6. Abrupt increases in the area of the hexagon formed by a given oxygen type were always accompanied by the rupture of H-bonds involving the corresponding hydroxyl group, and vice versa. There were slight differences of just 1–2 Å^2^ between the areas of the hexagons formed by the same type of oxygens from different CDs, larger for O3 and O5 of the CD that was closer to the SDS head (CD1) and for the O2 of the CD that was further (CD2). In general, these areas were practically constant with time, mainly those obtained from O4 atoms. The O6 was an exception to this behavior, showing sharp variations of more than 40 Å^2^ between minimum and maximum areas. Such variations were related to the high amplitude of the movement of these atoms leading to the petal-shaped patterns shown in Figure 4.

### 3.2. Comparison between G_2 and Other Simulation Methods Using GROMOS and AMBER/GAFF Force Fields in the Bulk Aqueous Solution

The 2:1 complexes in the simulations using GROMOS with the HMR and the H2Q methods with both time steps (2 and 7 fs) were stable and provided indistinguishable water density and dipolar moment profiles compared to the G_2 method (see Figures S2–S5 and Figures S8–S11). The projection of the motion of the different oxygen atoms along the trajectories was also identical for each group of simulations, indicating that the pathway and the amplitude of the internal motions of the CD groups and SDS molecule remained unaffected by the mass transfer or mass increase. This was also reflected in the indistinguishable areas of the hexagons formed by the oxygen atoms (Figures S12–S15). However, the results were quite different for the simulations using the AMBER/GAFF force field. In this case, the three high–low–high-density water layers were also visible, together with the dipole moment vector field of water molecules, but the motion of the CD oxygens was much more important and isotropic i.e., no privileged directions were observed in their motions. In Figure 3 and Figure S1, only the position of O5, O3, and O6 atoms were visible, O2 and O4 being shielded by them. Figures S6 and S7 show that the hexagonal shape water layer that was clear with the different parameterizations of GROMOS at 1–4 Å from the center of the structure was not visible when using the AMBER/GAFF force field. Additionally, the petal-shaped projection of the O6 motion was not present at all when using AMBER/GAFF. The three parameterizations of the AMBER/GAFF force field provided similar water densities and dipole moment profile, regardless of the method used to determine the partial charges and to parameterize the molecule (Figure 3, Figures S1, S6, and S7). For the AMBER/GAFF force field, the analysis of water density in concentric shells produced completely uniform patterns (not shown). There was also an important dipole moment vector field of water molecules around the structure of the complex, mainly near the SDS head, for the simulations with this force field. The areas formed by different types of oxygens using the AMBER/GAFF parameterization based on GPUs building blocks are shown in Figures S16 and S17. When using the AM1-BCC charges, the area of the hexagons formed by the six O6 atoms was significantly shorter than for the RESP charges. For this latter parameterization, the areas were more similar to those using the different versions of the GROMOS force field. In general, the areas obtained from AMBER/GAFF simulations were lower and fluctuated more than those using GROMOS.

### 3.3. H-Bond Analysis of Simulations in the Bulk Aqueous Solution

The number of intramolecular, CD-CD, and CD-water H-bonds were also equivalent for all the simulations using variations of the GROMOS force field (Table 1). In contrast, significant differences were observed for the corresponding H-bond lifetimes. In general, lifetimes of simulations using a time step of 7 fs were between 1.7 and 3.5 times larger than the equivalent simulations using a time step of 2 fs. The impact of the mass change at a constant time step in the H-bond lifetime was negligible for most H-bonds. The largest difference appeared for the lifetimes of the intramolecular H-bonds of the CD that was further from the SDS head. Even though the number of H-bonds was practically identical, their lifetimes for G_2 were 1640 ps while for HMR_2 was 1437 ps and for H2Q_2 was 1678 ps. It is interesting to see the different behavior of the interfacial hydroxyl groups (O2H2 and O3H3) between both CDs. The hydroxyl groups of CD1 formed significantly more H-bonds with water molecules than those of CD2. As a result, the intramolecular H-bonds of CD2 were twice more stable than those of CD1. This is reproducible with all methods based on the GROMOS and also on AMBER/GAFF force field, and it could be related to the higher order of water molecules around the hydroxyl groups of CD1 than of CD2 (see Figure 3, Figure 4 and Figure 5 and Figures S1–S8) induced by the closer proximity to the ionic SDS head. In general, the number of H-bonds obtained from any of the simulations using the AMBER/GAFF force field were very different from those obtained from the simulations based on the GROMOS force field. As shown in Table 1, for the simulations using AMBER/GAFF, the average number of CD1-CD2 intermolecular H-bonds was almost half (~9.4 vs. 5.5–5.8) of that using any of the GROMOS methods. The lifetime of these H-bonds obtained using the three AMBER/GAFF parameterizations was also significantly different to each other: 20.56, 37.61, and 18.21 ps for AMBER RESP, AMBER BB-RESP, and AMBER BB-AM1, respectively. In contrast, the number of intramolecular H-bonds was similar for all the AMBER/GAFF and GROMOS simulations, although the lifetime was much lower in AMBER than for the GROMOS parameterizations. The reduction in intermolecular CD1-CD2 H-bonds with AMBER/GAFF was compensated by a significant increase in the number of H-bonds between the O2H2 or O3H3 hydroxyls of both CDs and water molecules. The lifetime of such H-bonds was similar for AMBER/GAFF and for the GROMOS parameterizations. The O5 atom also participated in a larger number of H-bonds with water when using AMBER/GAFF than GROMOS, although the corresponding lifetime was similar. The number of H-bonds involving O6 was similar for all the parameterizations, just slightly lower for AMBER/GAFF.

### 3.4. Simulations at the Water/Air Interface Using the Different Parameterizations of the GROMOS Force Field

**Figure 7:**
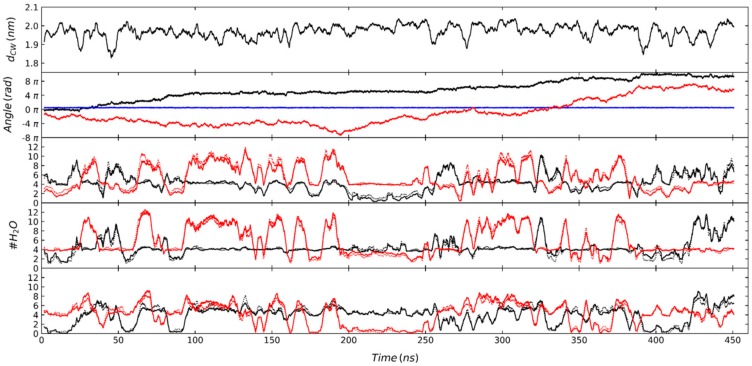
Distance between the center of the O4 atoms of the two CDs in the α-CD_2_SDS_1_ structure and the center of mass of the water molecules (first row). Yaw (red), pitch (blue), and roll (black) angles of the 2:1 complex as defined in Figure 2 with respect to the water/air interface (second row). Number of water molecules at less than 3 Å of any atom of the CD that is closer (solid lines) and further (dashed lines) to the SDS head in the complex (third to fifth rows). Rows 3, 4, and 5 represent GPU units {1,4}, {2,5}, and {3,6} for one CD (black lines), respectively, and the corresponding GPU units (i.e., those in the same generatrix of the nanocylinder-shaped structure) of the opposite CD (red lines). This plot results from the analysis of a MD trajectory using the G_2 parameterization method in the presence of a water/air interface. Equivalent plots for the rest of the studied parameterization methods are shown in the Supplementary Materials.

The α-CD_2_SDS_1_ complex was quickly adsorbed in the simulations where the interface was present, and it remained “floating” with the symmetry axis parallel to the water surface for the rest of the trajectories. A few exceptions to this behavior took place in some replicas of our simulations, where the structure rotated 90° and remained close to the interface with the symmetry axis perpendicular to the water surface. This behavior does not seem to depend on the different variations of the simulation method based on GROMOS and it is quite general for this force field, but it eventually appeared in some of the trajectories. An example of this behavior is shown in Figure S36 for the H2Q method with a time step of 2 fs, where the rotation took place at approximately 430 ns. The impact of the rotation of the structure was perfectly clear in the distance between the center of mass of the structure and that of the water molecules in the simulation box as well as in the pitch, yaw, and roll angles (see definition in Figure 2). The pitch angle went to 90° and the yaw and roll angles became much noisier (Figure S36). Notably, the hydration level of the different GPU residues after the rotation was lower than the maximum values reached by some residues when the structure was parallel to the interface. The possible presence of these alternative orientations for the complex (parallel and perpendicular to the interface) has been discussed in the past [23] using a battery of experimental data. Our simulations clearly indicate that the most probable orientation is that with the symmetry axis parallel to the surface but the rotation that appears in some simulations suggests that it could also be a marginal population of the perpendicular orientation. When the orientation of the complex was parallel to the surface, the level of water at which the 2:1 structure floats fluctuated significantly (± 3 Å), as indicated by the distance between the center of mass of the water molecules and that of the 2:1 complex (Figure 7, first row). The pitch angle was very small in all cases, indicating that the α-CD_2_SDS_1_ complex was really parallel to the water surface and it did not fluctuate significantly. The yaw angle changed slowly, although it did not seem to have a unidirectional rotation. However, the roll angle exhibited a clear trend to increase in the trajectory shown in Figure 7, completing more than four full turns after 450 ns. A similar increase in the roll angle can be seen in Figure S34 for one of the replicas of the HMR method with a time step of 2 fs and, to a lesser extent, in the H2Q simulation with time step of 7 fs shown in Figure S37. For the HMR_7 simulation shown in Figure S35, the average roll angle also increased although the structure only rotated 1.5 turns after 500 ns, while in the simulation shown in Figure S36, corresponding to the H2Q method with a time step of 2 fs, the rotation was small and it took place in the opposite direction. The anisotropic motion of the O6 atoms might favor the rotation of the roll angle in a single direction, although this could be shielded by other coupled movements. Actually, it is interesting to observe the orientation change of the α-CD_2_SDS_1_ complex at the interface, from parallel to perpendicular, which took place in the simulation where the roll angle decreased. The hydration level of the different GPU rings in the two CDs showed an interesting behavior with different patterns along the trajectories (see Figure 7 and Figures S34–S37, rows 3–5). For some periods, the hydration of most residues was quite high and fluctuated (around 50 ns in Figure 7) while for other periods the level of hydration of all residues was significantly lower (long period around 225 ns in Figure 7). The periods corresponding to low hydration seem to correlate with the regions where the distance between the complex and the center of mass of the water was larger and relatively stable, while the regions with high fluctuations seem to correspond to those where the complex was more buried in the water phase (shorter distance to the center of mass of the water molecules). Notably, the level of hydration in all residues was relatively low after the 90° rotation in the trajectory shown in Figure S36, even though the structure was less exposed to the air in that case.

**Figure 8:**
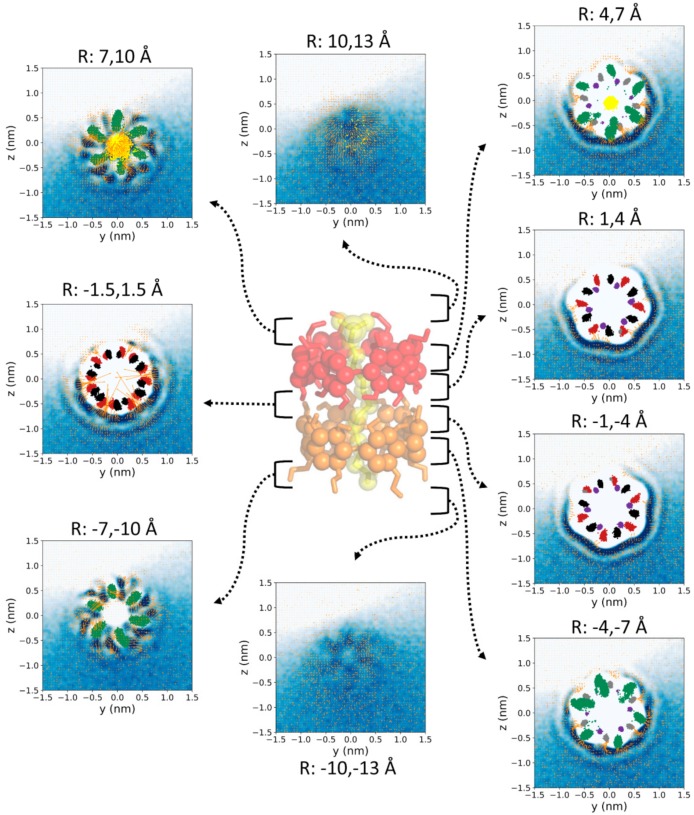
Water density (blue–white gradient from highest to lowest) and average water dipole moment (orange vectors) around the α-CD_2_SDS_1_ structure at the water/air interface for a trajectory generated using the G_2 method (original GROMOS 54a7 force field with 2 fs time step). Nine 3 Å width slices perpendicular to the symmetry axis of the complex were considered. The position of each slice is indicated by the brackets and arrows. The O2 (red), O3 (black), O4 (purple), O5 (grey), and O6 (green) atoms of the two CDs comprising the complex, together with the sulfur atom of the SDS molecule (yellow), are also represented. A selection of the last 10,000 frames of one trajectory with the complex aligned to the O4 atoms, corresponding to the frames where the GPU number 1 of the first CD has the minimum hydration, were employed for these calculations. A resolution of 0.5 × 0.5 Å^2^ in the YZ plane was considered to generate each plot. Equivalent plots for the rest of the studied parameterization methods are shown in the Supplementary Materials.

**Figure 9:**
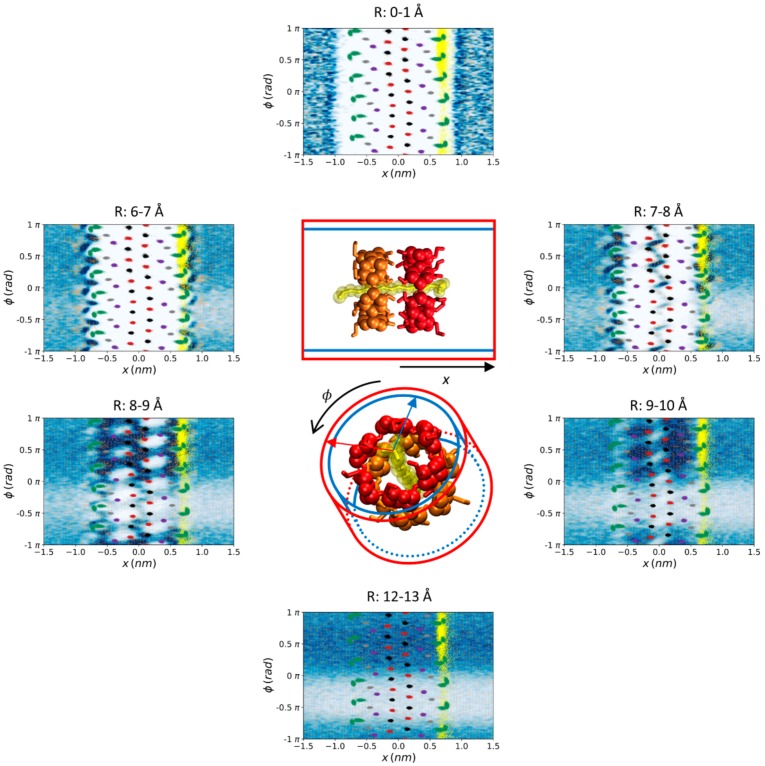
Water density (blue–white gradient from highest to lowest) and average water dipole moment (orange vectors) around the α-CD_2_SDS_1_ structure at the water/air interface for a trajectory generated using the G_2 method (original GROMOS 54a7 force field with 2 fs time step). Six 1 Å thick coaxial shells centered in the symmetry axis of the complex were considered and cylindrical coordinates are employed, for clarity. The distances to the symmetry axis corresponding to each shell is indicated in the figure. The O2 (red), O3 (black), O4 (purple), O5 (grey), and O6 (green) atoms of the two CDs comprising the complex, together with the sulfur atom of the SDS molecule (yellow), are also represented. The last 10,000 frames of one trajectory with the complex aligned to the O4 atoms were employed for these calculations. A resolution of 0.5 × 0.5 Å^2^ in the φx plane was considered to generate each plot. Equivalent plots for the rest of the studied parameterization methods are shown in the Supplementary Materials.

For the calculation of the density maps, only the frames corresponding to the minimum hydration level of each GPU group were selected, assuming that the rings with the minimum hydration level for a given frame are orientated towards the air. The density maps were similar to those corresponding to the complex in the bulk solution, but in this case, the exposition to the air left a region completely dried. The complementary density maps are shown in Figure 3 (right-top) as well as in Figure 8 and Figure 9. Figure 3 displays the comparison between the water density profile in the bulk solution and that at the interface. Upon adsorption, a significant number of highly packed water molecules were released, which could provide an entropic contribution to the adsorption. Notably, the high orientation of the water molecules around the complex also seemed to decrease, as revealed by the shorter dipole moment vector field shown around the complex structure (Figure 3, Figure 8 and Figure 9, Figures S18–S21, and Figures S24–S27). However, the wet region of the complex maintained the water structure around with a pattern that was quite similar to that in the bulk solution. The density maps shown as a function of the distance to the symmetry axis of the complex (Figure 3, right-top) reveals that the SDS head was slightly bent towards the water, since the density of sulfur in the dried region was slightly lower than that in the wet region.

The transition from a circular to hexagonal and again to circular density profile that had been observed in the bulk solution was also maintained at the interface despite the partial drying of the complex (Figure 8). Notably, even the petal-shaped density profiles induced by the particular movement of the O6 atoms were clearly seen in the presence of air/water interface (Figure 8). This suggests that just a few water molecules were required to avoid the loss of symmetry around the complex caused by the movement of the O6 groups. The complementary analysis shown in Figure 9 reveals that a thin layer of water appeared at a radius from the center of 7–8 Å, just between the O2 and O3 atoms, at the interface between both CDs, as happened in the case of the bulk simulations. However, even at this distance, the layer of waters was not completely uniform.

**Figure 10:**
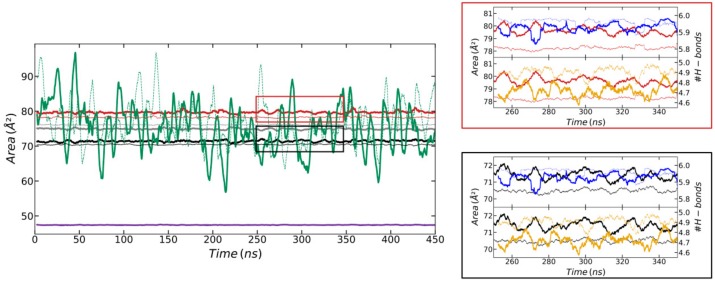
Area of the hexagons formed by the six O2 (red), O3 (black), O4 (purple), O5 (grey), and O6 (green) atoms of the CD molecules that are closer (solid lines) and further (dashed lines) from the SDS head. This plot corresponds to the simulation of the α-CD_2_SDS_1_ structure at the water/air interface for a trajectory generated using the G_2 method (original GROMOS 54a7 force field with 2 fs time step). Equivalent plots for the rest of the studied parameterization methods are shown in the Supplementary Materials.

The area of the hexagons formed by the six O2, O3, O4, or O5 atoms for the simulation at the interface were similar to those corresponding to the simulation in the bulk solution (Figure 10 and Figures S28–S31). The O6 again presented strong variations between the minimum and the maximum area. However, the amplitudes of the motion of these atoms leading to the petal-shaped patterns shown in Figure 9 were now a bit decreased in comparison to the results in the bulk (Figure 6 and Figures S12–S15).

### 3.5. H-Bond Analysis of Simulations at the Water/Air Interfaces Using the Different Parameterizations of the GROMOS Force Field

As in the case of the bulk solution, the number of intramolecular, CD-CD, and CD-water H-bonds were also equivalent for all the simulation methods (Table 2). In general, the average number of H-bonds seemed to be higher at the interface than in the bulk solution, although the discrepancies were not large. Again, important differences were observed in the H-bond lifetimes with all simulation methods. The most significant variation was observed between the lifetime of intramolecular H-bonds of CD1 vs. the same contribution in CD2, as in the case of the simulations in the bulk solution. That difference was attributed to the long-range effect of the charged SDS head, which induced a significant dipolar moment in the water molecules around CD1 and favored the interaction the O2-H2 and O3-H3 of this CD with water, compared to the equivalent interactions of CD2. In turn, the intramolecular H-bonds between O2-H2 and O3-H3 of CD2 were more stable than those for CD1. In this case, the relative difference is not as large as in the case of the simulations in the bulk solution, which might be due to the significant decrease in the dipolar moment of water around the complex in the bulk solution compared to the simulations at the interface. For the simulation using G_2, the lifetime values of the intramolecular H-bonds of CD1 and CD2 in aqueous solution were ~852 ps and ~1640 ps, respectively, while the equivalent interactions at the interface were ~1248 ps and ~1989 ps. In contrast with the simulations in the bulk aqueous solution, the presence of O4-water H-bonds was significant, although relatively low, at the interface. The H-bond pattern, completely reproducible with all the tested simulation methods using GROMOS (considering the correction factor observed in the simulations with a 7 fs time step), illustrates the significant increase in the number and stability of H-bonds from bulk to interface as well as the differences between both CDs in the supramolecular complex.

For the simulations at the interface, the distance between the center of mass of the simulation box and that of the complex, the roll, yaw, and pitch angles, the profile of the number of water molecules for each glucopyranoside ring as a function of time, and the area of the hexagons formed by different groups of oxygen atoms were also indistinguishable using the different GROMOS simulation methods and time steps of 2 and 7 fs (see Supplementary Materials). The number of intermolecular CD-CD and CD-water H-bonds were also equivalent between the two groups of simulations. As in the case of the simulations in the bulk solution, only the H-bond lifetimes were significantly increased. This indicates that both the HMR and the H2Q methods lead to identical structural results also in the interface and that the internal relative movements of the solute atomic groups are also identical.

### 3.6. Analysis of Simulations Using the AMBER Force Field in the Presence of the Water/Air Interfaces

Using the AMBER force field, the α-CD_2_SDS_1_ complex did not significantly adsorb at the interface and so the water density analysis and the determination of H-bonds for comparison with the simulations based on the GROMOS force field cannot be properly done. The complex can be observed very close to the interface at times during the MD trajectories due to its diffusion but it was not stable enough to perform a reasonable characterization (see Figures S38 and S39). Interestingly, when it was close to the interface, it appeared with the symmetry axis in the perpendicular orientation. The density profile corresponding to these simulations is shown in Figure 3 (right-bottom) and Figure S1 but the water/air interface cannot be appreciated because the complex does not stay at the interface for a significant time. Thus, the water density profile, dipole moment vector field, and H-bond pattern (Table 2) of these trajectories were quite similar to those obtained for the same force field in the bulk solution.

## 4. Discussion

The system selected for this study, a α-CD_2_SDS_1_ complex, proved to be extremely sensitive to different factors including temperature, concentration range, and the presence of water/air interface. A number of different experimental methods including calorimetry, surface tension, rheology, neutron reflectometry, ellipsometry, and several microscopies were employed to characterize its behavior under different conditions [1,5,6,23]. Several studies were also performed by using β-CD_2_SDS_1_ instead of α-CD_2_SDS_1_ complexes [4,27,28], as well as other similar structures that provide interesting self-assembly patterns in the bulk solution and at interfaces [29,30]. However, the driving forces responsible for those events and arrangements are not clear. The connection between atomistic and macroscopic level information might be employed to design new applications as well as to optimize the already established employments of these systems. CDs are well suited for atomic resolution computational studies due to their small size and low number of degrees of freedom. A number of docking and computational studies involving CDs have been published [10,11,12,26] but the use of these tools is much less extended for these molecules than in protein, peptide, or lipid systems. Thus, the proposal and validation of reliable and efficient computational methods for CD systems is expected to contribute to acquire a better knowledge able to complement the interpretation of wet-lab measurements.

Here, we performed MD simulations of α-CD_2_SDS_1_ complexes in the bulk aqueous solution as well as in the presence of water/air interfaces using five different parameterizations of the GROMOS force field and three different parameterizations of the AMBER/GAFF force field. The different parameterizations tested using GROMOS were aimed at optimizing the sampling, minimizing the use of computational resources. For this, we took advantage of manipulating the highest frequency motions, which in any molecule corresponds to the lightest atoms, i.e., the Hs. The time step employed in MD simulations for the integration of the motion equation is necessarily limited by these fastest degrees of freedom. For this reason, a time step of 2 fs is employed in most atomic force fields. Different methods have been proposed and tested to overcome this limitation. We decided to compare MD simulations using the standard GROMOS 54a7 force field (G_2), simulations transferring 3 Da of mass to the H atoms from the bound heavy atoms with 2 fs (HMR_2) and 7 fs (HMR_7) time steps, and simulations increasing the mass of H atoms from 1 to 4 Da with the same time steps (H2Q_2 and H2Q_7). Preliminary work performed in our lab indicated that simulations using AMBER behave significantly different from those using GROMOS for CD systems. Thus, we decided to test three different parameterizations of this force field, trying to explain the source of such discrepancy with experiments as well as with results using GROMOS. Simulations of the α-CD_2_SDS_1_ complex using the eight different parameterizations were performed in the bulk aqueous solution and also in the presence of water/air interfaces. As expected, the mass increase of H in the GROMACS parameterization using the H2Q method slows down the motion of these atoms, thus producing stable trajectories even with a time step of 7 fs. It has been said that the application of this method could negatively contribute to the sampling when applied to AA force fields, even if the time step is larger, due to slower diffusion that the resulting heavier molecules would exhibit [22]. However, for UA force fields, the impact of the mass increase is much lower. This can be illustrated by a simple calculation: by increasing the mass of all H atoms of the α-CD molecules in 3 Da, the total mass increase of the molecule would be 18.5% in an AA representation and just 5.5% when using the GROMOS force field. The difference is even larger for the SDS molecule since the total mass increase using an AA force field would be 26% while the lack of polar H atoms in this molecule implies that the mass does not change at all upon the application of this method using the GROMOS force field. In addition to the latter argument, the mass increase just in the polar H atoms for the UA force fields can also be justified based on the experimental fact that only the polar H atoms are spontaneously deuterated (thus doubling their mass) when they are solvated in deuterated water [1,23]. This experimental increase of mass just in the α-CD polar Hs maintains the stoichiometry, enthalpies, and equilibrium constants of the binding with SDS molecules, as revealed by ITC measurements performed using different isotopic contrasts, namely α-CD with hydrogenated and deuterated SDS in deuterated and non-deuterated water [23]. Our proposal of increasing the H mass from 1 to 4 Da is based on the hypothesis that the use of quadium instead of deuterium (another mass increase by a factor of 2) also maintains the thermodynamic behavior of this system. Note that in this work, we did not change the parameterization of the water, which is equivalent to using one of the several possible isotopic contrasts. Our results provide almost indistinguishable results using the different GROMOS parameterizations, regardless of the mass of the H atoms. The highly specific density maps as well as the water dipole moment vector field were absolutely reproducible in all the details, both in the bulk solution and at the interface. It is worth to mention that the application of the mass increase just for the polar H atoms in AA force fields would not represent any advantage since the presence of nonpolar light H atoms would not make possible to increase the time step.

The simulations using the AMBER/GAFF force field provided completely different results, illustrating the high sensitivity of this system. In all the simulations performed in the presence of water/air interface using the different parameterizations of the GROMOS force field, the complex quickly adsorbed with the symmetry axis parallel to the surface. The perpendicular orientation was also observed in some marginal cases of this force field. Again, these results do not seem to depend on the method employed to deal with the mass of the H atoms nor on the time step. However, for the simulations performed using the AMBER force field, the affinity of the complex by the interface is negligible. In these simulations, the complex does not adsorb to the interface at all or, when it is adsorbed, the orientation is perpendicular to the surface (see Results section). The source of this discrepancy seems to be in the low number of intramolecular CD1-CD2 H-bonds in the complex for this force field, almost half the amount observed for all the parameterizations using GROMOS. Since the strength of the H-bonds critically depends on the partial charges, the three AMBER/GAFF parameterizations tested were performed in completely different ways, namely (*i*) using the *antechamber* tool with RESP charges for the whole CD; (*ii*) using the antechamber tool with RESP charges to parameterize the methylated GPUs and then manually pasting them together to build the topology of the whole CD; and (*iii*) same as (*ii*) but using AM1-BCC charges. The first strategy introduces an artificial asymmetry in the charge distribution for chemically equivalent groups so the other two approaches, based on the building block strategy, could be considered as more reliable. The results coming from the three different AMBER/GAFF parameterizations are significantly different to each other but they match in the number of intramolecular CD1-CD2 H-bonds in the complex, approximately half of that observed for all the simulations using GROMOS, as well as in the larger number of H-bonds between the hydroxyls involving O2 and O3 with the solvent molecules. Thus, the differences between the two force fields rely on the intrinsic force field parameters (bond and angle constants) more than in the partial charges. The impact of this specific difference between both force fields is extremely serious for this system due to its high sensitivity to the presence of the water/air interface. As a result, the α-CD_2_SDS_1_ complexes exhibit no significant affinity to the interface with AMBER/GAFF, in contrast to experimental results and with the simulations using the different parameterizations of the GROMOS force field. These results indicate that the AMBER/GAFF force field does not describe the behavior of this system in a reliable way, regardless of the method employed to get the partial charges. Additionally, the simulations using GROMOS suggest a possible driving force for the adsorption of the CD-based complexes. The high specificity of the water distribution around the complex, like a well-fitted dress over the structure, with a relatively low number of H-bonds between the CDs and the water, play the role of a hydrophobic solvent shell that is partially released upon adsorption to the interface. This is expected to provide a favorable entropic contribution associated to this process. For the simulations using AMBER/GAFF, there is also a high-density water shell around the complex, but it is much less specific than in the case of GROMOS and the number of H-bonds with the CDs is significantly higher in the case of AMBER/GAFF. Thus, the force field parameters, providing a lower number of intermolecular CD1-CD2 H-bonds, lead to an important qualitative difference since they determine whether or not the structure adsorb to the interface. As stated above, there are conclusive experimental evidences on the adsorption of α-CD_2_SDS_1_ complexes at the water/air interface, but there is also an open discussion on its orientation with respect to the surface plane [23]. A combination of atomic force microscopy, neutron reflectometry, and ellipsometry experiments suggests that the symmetry axis of the complex is parallel to such surface [6,23] but there are not conclusive evidences for this. Our simulations using different parameterizations of GROMOS also indicate that the most probable orientation is the parallel one, but the perpendicular orientation also appeared marginally in some simulations. This suggests that both orientations might live together at the interface with different probability.

Some of our results could be extrapolated to other similar systems such as β-CD_2_SDS_1_ complexes, which exhibit an extremely interesting aggregation behavior in the bulk aqueous solution [4]. The driving force for the self-aggregation of such complexes and for the stabilization of the resulting mesoscopic structures has been discussed [27,28] but the arguments are not conclusive. Simulations of this complex in aqueous solution, using the H2Q_7 method, are expected to reproduce the aggregation pattern with atomic resolution, at least at a modest size scale of 10–15 nm. Additionally, the aggregation of α-CD_2_SDS_1_ complexes in the bulk solution has not been experimentally studied and it is expected to also produce interesting patterns with potential practical applications.

## 5. Conclusions

In the present work, we provide a detailed analysis of the behavior of the water around α-CD_2_SDS_1_ complexes in the bulk aqueous solution and at the water/air interface. For the simulations using different parameterizations of the GROMOS force field, highly specific density patterns and dipole moment vector fields are observed. The internal movement of the different groups within the complex are described and connected with the profile of specific regions with high water density. Especially interesting is the trajectory of the O6 atoms, showing an anisotropic petal-shaped profile probably due to the chirality of the GPU rings of the CD molecules. Such a movement seems to provoke a unidirectional rotation around the symmetry axis of the complex when it is adsorbed at the water/air interface. The adsorption of the complex is observed to be very quick (just a few ns) and the complex remains stable with its symmetry axis parallel to the water surface for the rest of the trajectory. However, a significant oscillation of the structure (~3 Å amplitude) with respect to the water surface is observed. The adsorption of the complex allows releasing of a significant number of highly packaged water molecules as well as a partial disorder of the solvent. This is translated in a decrease of the local sources and sinks of the dipolar moment vector field. Such release of packed water molecules and the associated decrease of solvent order could represent an entropic driving force for the adsorption of the complexes and also for self-assembly processes, since the aggregation of several 2:1 structures is also expected to allow releasing of neighboring water molecules.

In addition to illustrate the fanciful behavior of water molecules around the α-CD_2_SDS_1_ complex in water solution and at the water/air interface, the same highly specific analysis for different variations of the GROMOS force field that allow to significantly increase the sampling are tested, thus optimizing the use of computational resources. The HMR method was already proposed 20 years ago and it has been employed in a number of papers with reasonably good results. Here, we propose and test the use of what we call the H2Q method (hydrogen to quadium replacement) based on the experimental observation that deuterated CDs just in the polar Hs lead to the same stoichiometry and thermodynamic parameters than hydrogenated CDs [23] and on the assumption that the replacement of deuterium by quadium also provides similar results. This allows significantly increasing the integration time step, thus speeding up MD simulations. The results obtained using both methods are found to be identical to those using the original force field, except in the lifetime of the H-bonds involving the modified H atoms.

In contrast to wet-lab experiments and to the results obtained using different variations of the GROMOS force field, the α-CD_2_SDS_1_ complex does not adsorb to the water/air interface when using different parameterizations of the α-CD for the AMBER/GAFF. This suggests that the latter force field is not well suited for simulations of the studied CD systems.

Overall, this paper is expected to contribute to the understanding of the behavior of supramolecular complexes based on CDs, revealing the importance of the solvent molecules as well as of the chirality of the glucopyranoside rings. Additionally, the HMR and the H2Q methods to optimize the sampling of the simulations provided exceptionally good results, while the AMBER/GAFF force field failed even at qualitative level. Our results provide an explanation for the formation and stability of several structures that have significant practical applications, including the design of smart films with specific mechanical properties for functional coatings, the synthesis of dielectric actuators based on cyclodextrin complexes (http://www.asmi.jp/en/tec), artificial cell membranes, the so-called cyclodextrinosomes [29,30], and other recently discovered compartmentalized structures that will eventually lead to new applications [4]. All these new materials and structures were experimentally observed and characterized, but their behavior at atomic scale is still unknown. We aim to contribute to fill this gap between fundamental knowledge and practical applications. Additionally, the studied α-CD_2_SDS_1_ complex proved to be highly sensitive to the force field, so we propose to use it in order to test new parameters for computational simulations.

## Figures and Tables

**Table 1:** Average number of different H-bonds contributions and simulation methods together with the corresponding lifetimes in ps (in brackets) for the different molecular dynamics (MD) simulations in the bulk solution.

Donor/Acceptor	G_2	HMR_2	HMR_7	H2Q_2	H2Q_7	AMBER RESP	AMBER BB-RESP	AMBER BB-AM1
CD1-CD2	9.37 (41.01)	9.37 (41.00)	9.47 (148.42)	9.40 (41.75)	9.42 (145.47)	5.87 (20.56)	5.45 (37.61)	4.85 (18.21)
O2H2 (CD1)-O3H3 (CD2)	4.55 (37.78)	4.52 (37.87)	4.60 (138.35)	4.56 (38.63)	4.59 (137.23)	2.58 (21.42)	1.57 (41.36)	1.96 (18.64)
O2H2 (CD2)- O3H3 (CD1)	4.76 (44.37)	4.74 (43.93)	4.77 (158.85)	4.74 (44.78)	4.74 (153.70)	2.60 (21.51)	1.59 (42.60)	1.93 (18.81)
CD1-CD1	5.87 (852)	5.82 (856)	5.86 (2624)	5.85 (849)	5.87 (2696)	5.80 (250.07)	5.46 (145.83)	5.73 (210.60)
O3H3 (CD1)- O2H2 (CD1)	5.87 (856)	5.82 (859)	5.86 (2631)	5.85 (853)	5.87 (2701)	5.77 (291.50)	5.43 (155.73)	5.71 (227.72)
CD1-SDS	1.48 (52.26)	1.35 (53.58)	1.39 (93.65)	1.55 (52.70)	1.41 (89.89)	2.02 (17.98)	0.89 (19.63)	2.55 (21.00)
CD2-CD2	5.94 (1640)	5.93 (1437)	5.92 (4484)	5.92 (1678)	5.92 (4586)	5.89 (310.17)	5.66 (209.38)	5.81 (233.32)
O3H3 (CD2)- O2H2 (CD2)	5.94 (1645)	5.93 (1444)	5.92 (4494)	5.92 (1678)	5.92 (4607)	5.84 (400.60)	5.64 (223.52)	5.76 (271.77)
O2H2 (CD1)-water	2.32 (6.90)	2.37 (7.03)	2.36 (19.47)	2.34 (6.95)	2.33 (19.35)	3.45 (7.49)	4.41 (9.72)	3.14 (7.94)
O2H2 (CD2)-water	1.97 (6.45)	1.95 (6.54)	2.01 (19.09)	1.99 (6.65)	2.01 (19.11)	3.18 (7.24)	3.91 (9.45)	2.89 (7.85)
O3H3 (CD1)-water	1.40 (6.23)	1.43 (6.35)	1.38 (18.64)	1.39 (6.24)	1.39 (18.73)	2.98 (6.99)	3.29 (8.39)	3.26 (7.45)
O3H3 (CD2)-water	1.19 (5.87)	1.17 (5.82)	1.18 (18.30)	1.21 (5.85)	1.20 (18.43)	2.72	2.98 (8.27)	3.00 (7.48)
						(7.06)		
O5 (CD1)-water	1.93 (6.60)	1.93 (6.62)	1.98 (18.79)	1.95 (6.75)	1.97 (18.77)	2.80 (6.50)	4.67 (9.43)	2.78 (6.80)
O5 (CD2)-water	1.63 (6.47)	1.60 (6.43)	1.64 (18.67)	1.62 (6.50)	1.62 (18.74)	2.42 (6.33)	4.40 (9.28)	2.32 (6.55)
O6H6 (CD2)-water	12.44 (14.49)	12.46 (14.76)	12.56 (27.73)	12.45 (14.82)	12.52 (28.27)	9.71 (7.48)	10.96 (7.96)	9.53 (7.69)
O6H6 (CD1)-water	11.59 (12.16)	11.68 (12.36)	11.73 (24.84)	11.50 (12.30)	11.72 (24.87)	10.72 (8.16)	11.31 (8.75)	10.99 (8.32)
SDS-water	3.69 (12.35)	3.74 (12.53)	3.74 (25.70)	3.63 (12.38)	3.72 (25.67)	3.53 (8.96)	3.75 (11.35)	3.37 (8.64)

**Table 2:** Average number of different H-bonds contributions and simulation methods together with the corresponding lifetimes in ps (in brackets) for the different simulations at the water/air interface.

Donor/Acceptor	G_2	HMR_2	HMR_7	H2Q_2	H2Q_7	AMBER RESP	AMBER BB-RESP	AMBER BB-AM1
CD1-CD2	9.64 (46.60)	9.65 (46.58)	9.71 (166.19)	9.61 (45.75)	9.67 (163.43)	5.87 (20.50)	5.40 (36.66)	4.86 (18.81)
O2H2 (CD1)-O3H3 (CD2)	4.71 (42.81)	4.72 (42.66)	4.76 (155.15)	4.69 (42.10)	4.70 (147.75)	2.55 (21.27)	1.61 (39.79)	1.98 (19.66)
O2H2 (CD2)- O3H3 (CD1)	4.90 (50.71)	4.91 (50.94)	4.93 (178.41)	4.89 (49.77)	4.93 (181.16)	2.64 (21.37)	1.54 (41.16)	1.98 (19.21)
CD1-CD1	5.91 (1248)	5.92 (1381)	5.93 (3852)	5.91 (1298)	5.90 (3662)	5.79 (244.78)	5.47 (151.57)	5.73 (208.11)
O3H3 (CD1)- O2H2 (CD1)	5.91 (1262)	5.92 (1398)	5.93 (3878)	5.91 (1311)	5.90 (3695)	5.76 (284.60)	5.45 (160.66)	5.71 (223.89)
CD1-SDS	2.09 (47.35)	1.89 (48.18)	1.99 (104.61)	2.05 (56.39)	2.16 (93.78)	1.96 (18.15)	1.01 (19.74)	2.25 (20.96)
CD2-CD2	5.96 (1989)	5.97 (2505)	5.96 (5624)	5.96 (2144)	5.97 (8040)	5.89 (318.85)	5.56 (182.09)	5.82 (251.10)
O3H3 (CD2)- O2H2 (CD2)	5.96 (2117)	5.96 (2632)	5.96 (5869)	5.96 (2307)	5.96 (8421)	5.84 (411.09)	5.54 (191.28)	5.78 (289.62)
O2H2 (CD1)-water	2.51 (6.17)	2.39 (6.19)	2.76 (18.49)	2.34 (6.22)	2.28 (18.77)	3.51 (7.42)	4.53	3.17 (7.79)
							(9.77)	
O2H2 (CD2)-water	2.24 (5.85)	2.16 (5.88)	2.52 (18.24)	2.08 (5.99)	2.03 (18.43)	3.23 (7.25)	3.97	3.00 (7.86)
							(9.35)	
O3H3 (CD1)-water	1.87 (5.62)	1.72 (5.65)	2.10 (18.04)	1.67 (5.68)	1.62 (18.24)	2.99 (7.03)	3.37	3.33 (7.52)
							(8.28)	
O3H3 (CD2)-water	1.72 (5.45)	1.61 (5.44)	1.99 (17.94)	1.51 (5.48)	1.49 (17.95)	2.79 (6.89)	2.99	3.07 (7.36)
							(8.23)	
O4 (CD1)-water	0.56 (5.10)	0.47 (5.09)	0.69 (17.75)	0.39 (5.09)	0.40 (17.53)	--	--	--
O4 (CD2)-water	0.55 (5.10)	0.50 (5.08)	0.68 (17.50)	0.44 (5.09)	0.40 (17.58)	--	--	--
O5 (CD1)-water	2.05 (6.07)	2.01 (6.13)	2.27 (18.26)	1.97 (6.10)	1.94 (18.41)	2.83 (6.48)	4.70	2.82 (6.84)
							(9.44)	
O5 (CD2)-water	1.82 (5.95)	1.75 (6.01)	1.98 (18.07)	1.68 (5.94)	1.68 (18.40)	2.46 (6.26)	4.46	2.37 (6.45)
							(9.07)	
O6H6 (CD2)-water	11.33 (11.89)	11.36 (12.13)	11.74 (24.99)	11.24 (12.12)	11.12 (25.27)	9.83 (7.48)	10.98 (7.90)	9.80 (7.65)
O6H6 (CD1)-water	12.79 (15.68)	12.70 (15.83)	12.95 (31.75)	12.74 (15.83)	12.65 (32.11)	10.89 (8.13)	11.55 (8.55)	11.17 (8.27)
SDS-water	3.49 (11.01)	3.59 (11.34)	3.61 (24.84)	3.42 (11.13)	3.34 (25.07)	3.62 (8.97)	3.66 (11.18)	3.62 (8.80)

## Supplementary Materials

The Supplementary Figures S1–S39 are available online at https://www.mdpi.com/2218-273X/10/3/431/s1.
