# Supplementary material for: Rings, Hexagons, Petals, and Dipolar Moment Sink-Sources: The Fanciful Behavior of Water around Cyclodextrin Complexes
Source: Biomolecules. 2020 Mar 10;10(3):431. doi: 10.3390/biom10030431 (PMC7175221; doi:10.3390/biom10030431)
Supplement: Supplementary file 1 [file biomolecules-10-00431-s001.pdf]

Supplementary material for

Rings, hexagons, petals and dipolar moment sink-sources: the fanciful behavior of water around cyclodextrin complexes

Pablo F. Garrido[1], Martín Calvelo[2], Rebeca García-Fandiño[2,*], Ángel Piñeiro[1,*]

[1] Departamento de Física de Aplicada, Facultade de Física, Universidade de Santiago de Compostela, E-15782 Santiago de Compostela, Spain

[2] Departamento de Química Orgánica, Center for Research in Biological Chemistry and Molecular Materials, Universidade de Santiago de Compostela, Campus Vida s/n, E-15782 Santiago de Compostela, Spain

* rebeca.garcia.fandino@usc.es; Angel.Pineiro@usc.es

Contents: Figures S1-S39

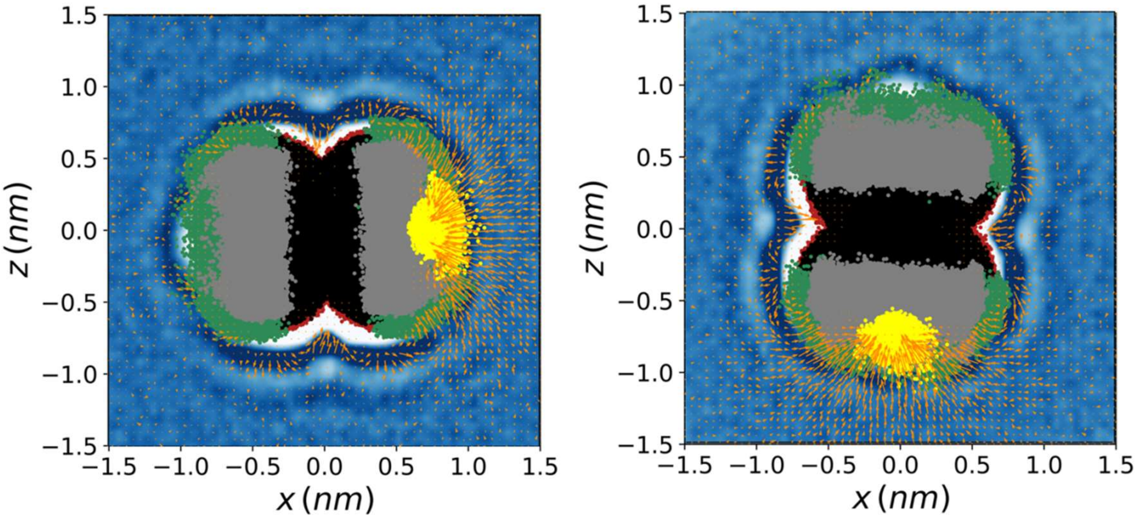

Figure S1.- Water density (blue-white gradient from highest to lowest) and average water dipole moment (orange vectors) around the α-CD_2SDS_1 complex at the interface (top) and in aqueous solution (bottom) for two trajectories generated using the AMBER force field with AM1-BCC charges. The O2 (red), O3 (black), O4 (purple), O5 (grey), and O6 (green) atoms of the two CDs consisting the complex, together with the sulfur atom of the SDS molecule (yellow), are also represented. The last 10000 frames of one trajectory with the complex aligned to the O4 atoms were employed for these calculations. A resolution of 0.5x0.5 $Å^2$ in the XZ plane for a 3 Å width slice perpendicular to the Y axis and centered in the symmetry axis of the complex was considered to generate the plot.

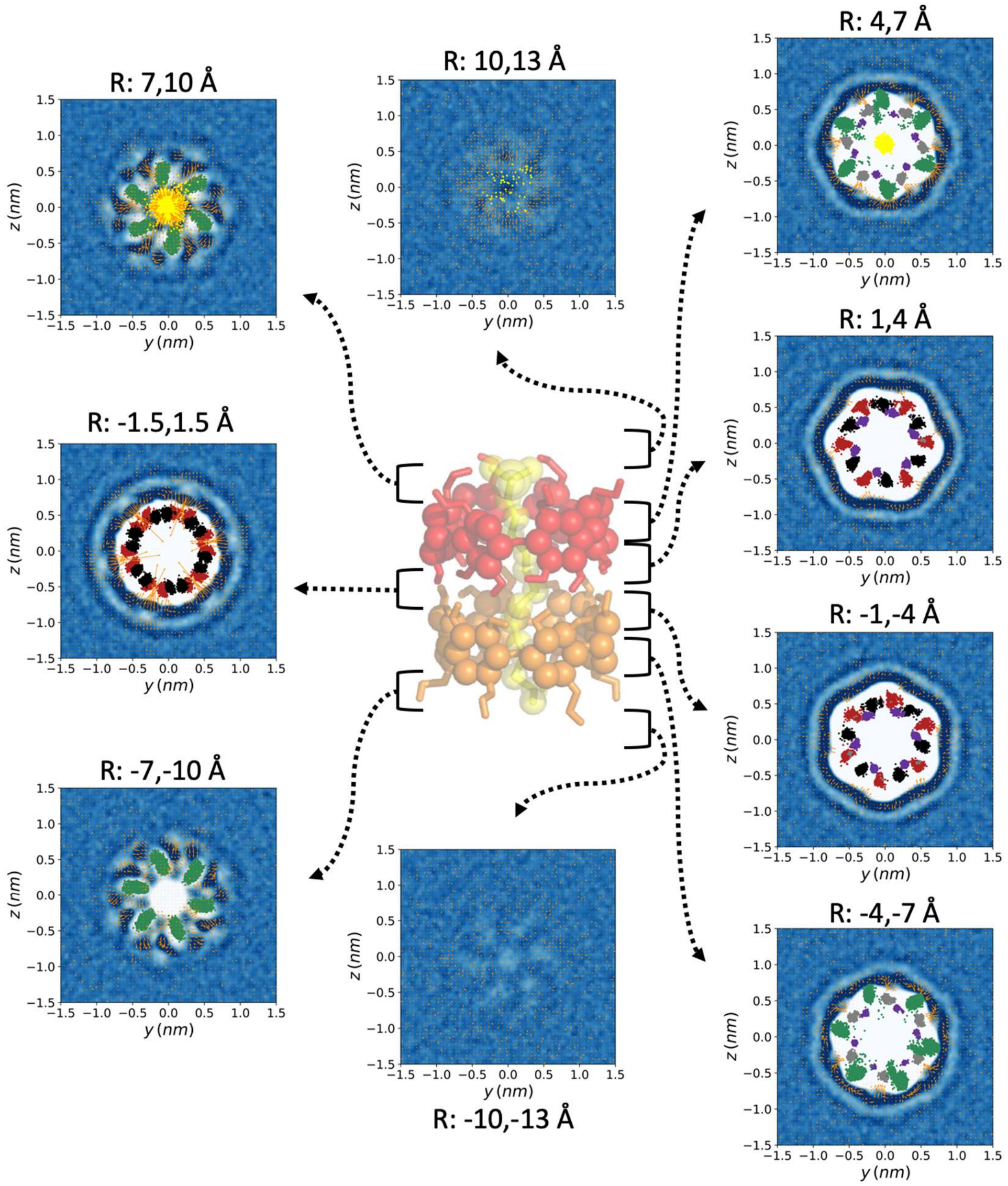

Figure S2.- Water density (blue-white gradient from highest to lowest) and average water dipole moment (orange vectors) around the **α-CD_2SDS_1 structure in aqueous solution** for a trajectory generated using the **HMR_2 method**. Nine 3 Å width slices perpendicular to the symmetry axis of the complex were considered. The position of each slice is indicated by the brackets and arrows. The O2 (red), O3 (black), O4 (purple), O5 (grey), and O6 (green) atoms of the two cyclodextrins consisting the complex, together with the sulfur atom of the SDS molecule (yellow), are also represented. The last 10000 frames of one trajectory with the complex aligned to the O4 atoms were employed for these calculations. A resolution of 0.5x0.5 $Å^2$ in the YZ plane was considered to generate each plot.

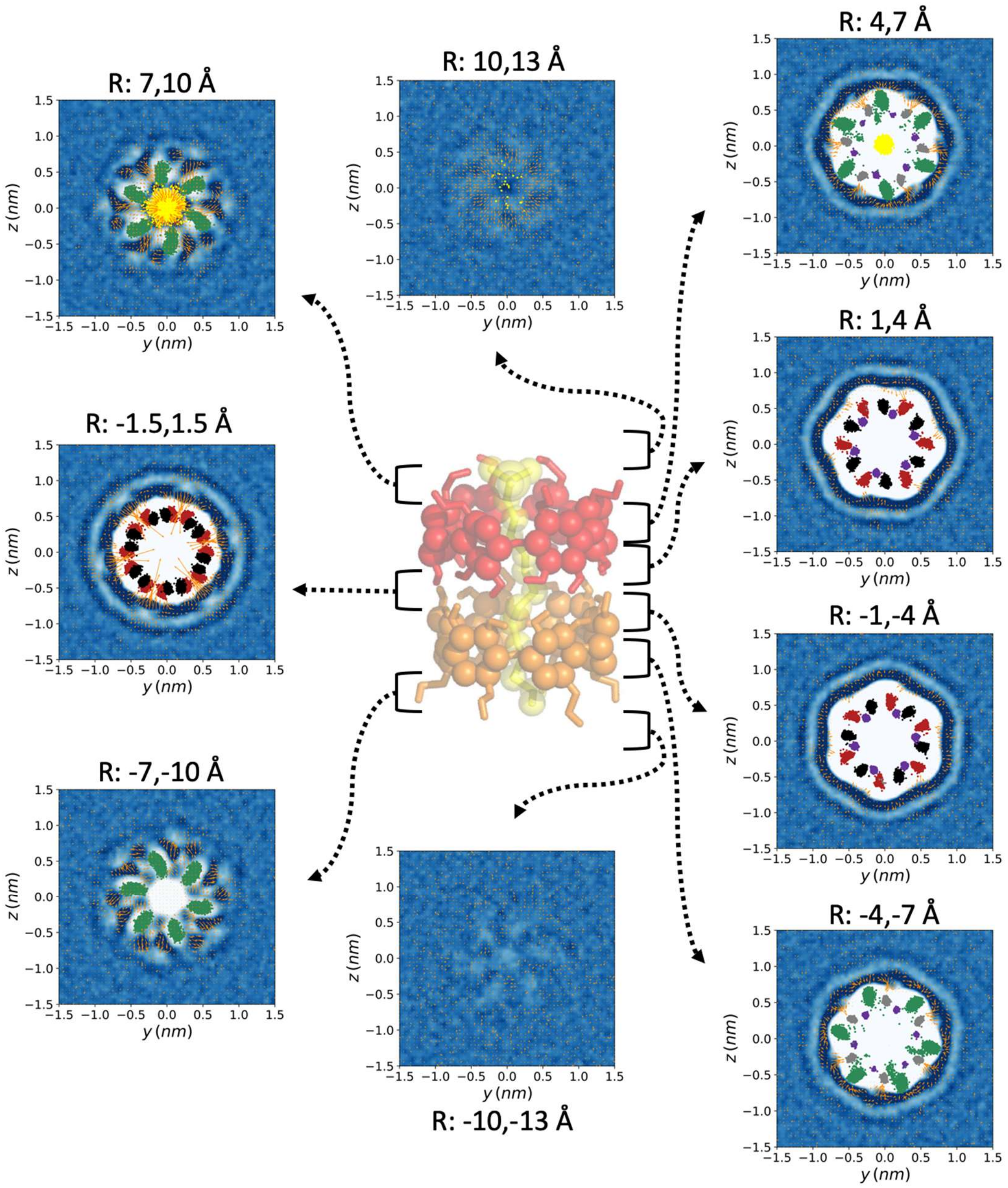

Figure S3.- Water density (blue-white gradient from highest to lowest) and average water dipole moment (orange vectors) around the **α-CD_2SDS_1 structure in aqueous solution** for a trajectory generated using the **HMR_7 method**. Nine 3 Å width slices perpendicular to the symmetry axis of the complex were considered. The position of each slice is indicated by the brackets and arrows. The O2 (red), O3 (black), O4 (purple), O5 (grey), and O6 (green) atoms of the two cyclodextrins consisting the complex, together with the sulfur atom of the SDS molecule (yellow), are also represented. The last 10000 frames of one trajectory with the complex aligned to the O4 atoms were employed for these calculations. A resolution of 0.5x0.5 $Å^2$ in the YZ plane was considered to generate each plot.

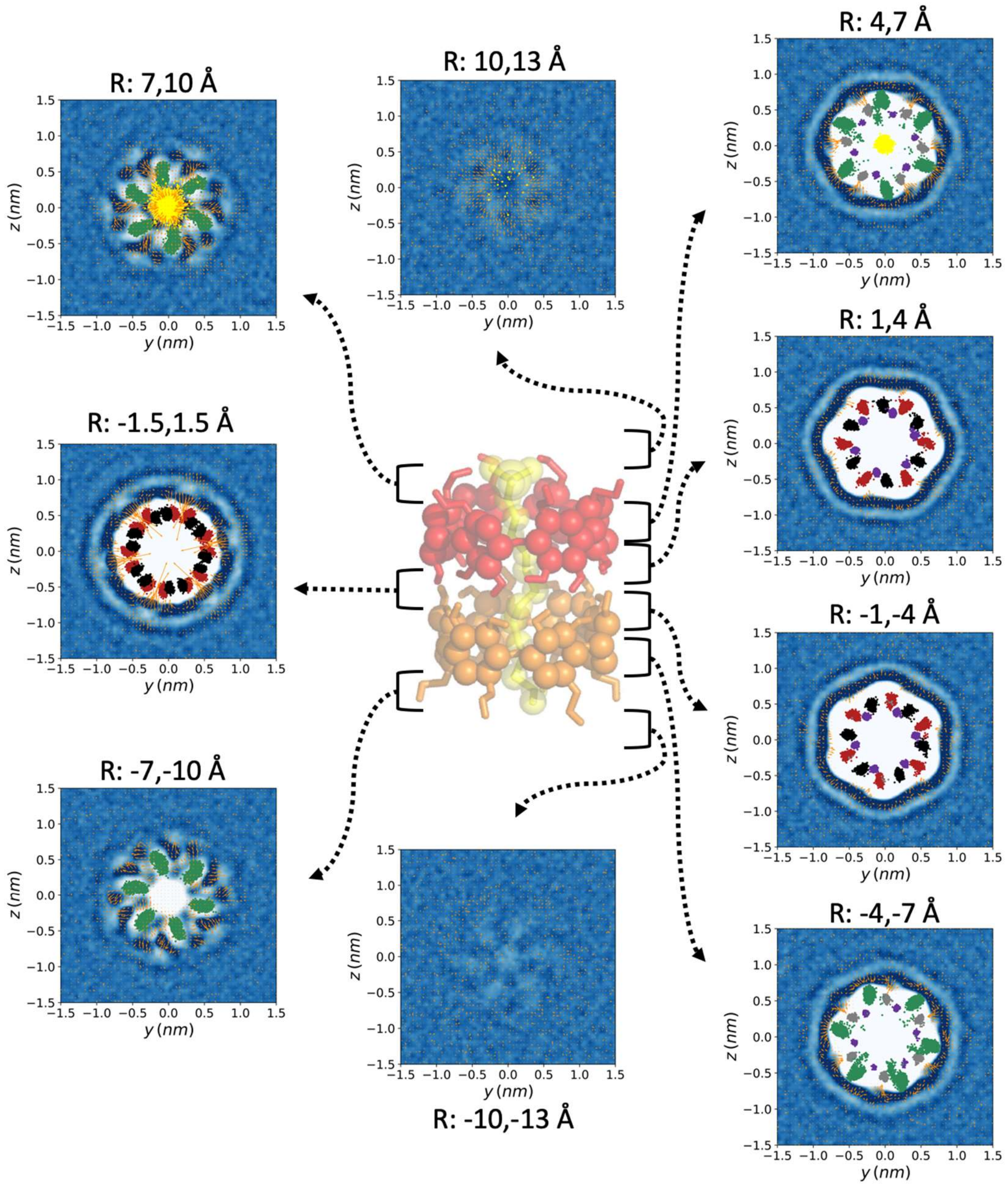

Figure S4.- Water density (blue-white gradient from highest to lowest) and average water dipole moment (orange vectors) around the **α-CD$_2$SDS$_1$ structure in aqueous solution** for a trajectory generated using the **H2Q_2 method**. Nine 3 Å width slices perpendicular to the symmetry axis of the complex were considered. The position of each slice is indicated by the brackets and arrows. The O2 (red), O3 (black), O4 (purple), O5 (grey), and O6 (green) atoms of the two cyclodextrins consisting the complex, together with the sulfur atom of the SDS molecule (yellow), are also represented. The last 10000 frames of one trajectory with the complex aligned to the O4 atoms were employed for these calculations. A resolution of 0.5x0.5 Å^2 in the YZ plane was considered to generate each plot.

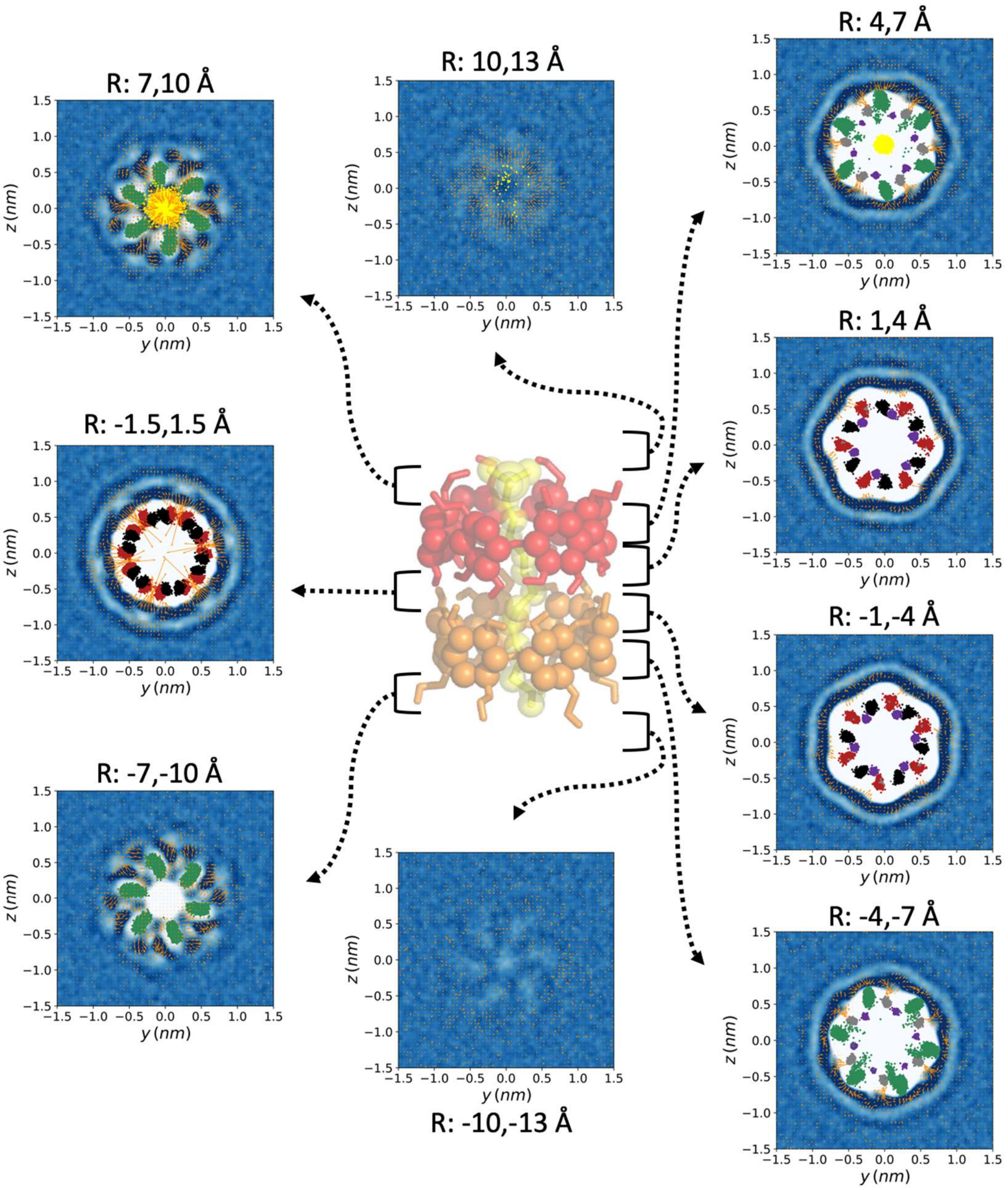

Figure S5.- Water density (blue-white gradient from highest to lowest) and average water dipole moment (orange vectors) around the **α-CD_2SDS_1 structure in aqueous solution** for a trajectory generated using the **H2Q_7 method**. Nine 3 Å width slices perpendicular to the symmetry axis of the complex were considered. The position of each slice is indicated by the brackets and arrows. The O2 (red), O3 (black), O4 (purple), O5 (grey), and O6 (green) atoms of the two cyclodextrins consisting the complex, together with the sulfur atom of the SDS molecule (yellow), are also represented. The last 10000 frames of one trajectory with the complex aligned to the O4 atoms were employed for these calculations. A resolution of 0.5x0.5 $Å^2$ in the YZ plane was considered to generate each plot.

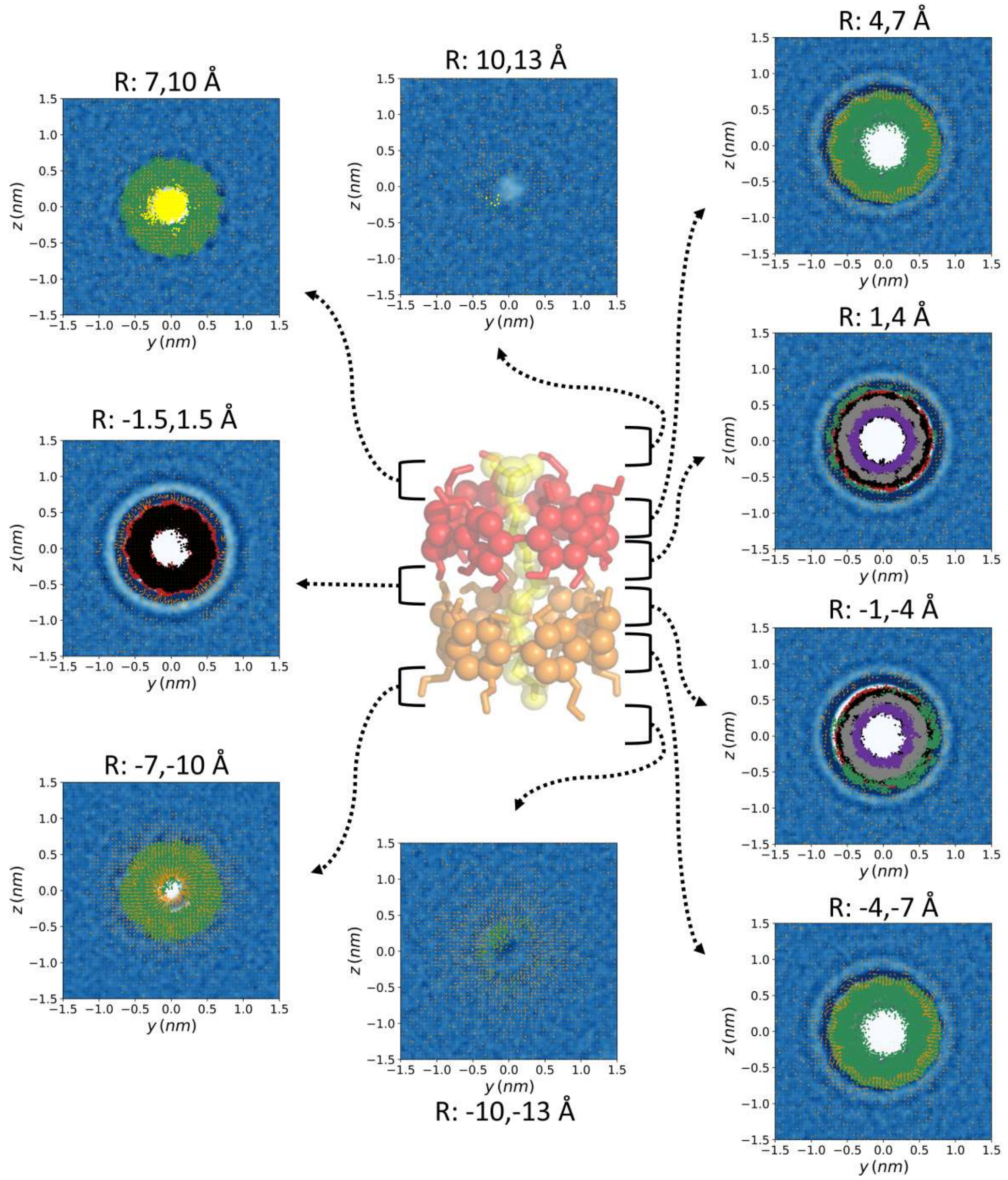

Figure S6.- Water density (blue-white gradient from highest to lowest) and average water dipole moment (orange vectors) around the **α-CD_2SDS_1 structure in aqueous solution** for a trajectory generated using the **AMBER BB-RESP method**. Nine 3 Å width slices perpendicular to the symmetry axis of the complex were considered. The position of each slice is indicated by the brackets and arrows. The O2 (red), O3 (black), O4 (purple), O5 (grey), and O6 (green) atoms of the two cyclodextrins consisting the complex, together with the sulfur atom of the SDS molecule (yellow), are also represented. The last 10000 frames of one trajectory with the complex aligned to the O4 atoms were employed for these calculations. A resolution of 0.5x0.5 $Å^2$ in the YZ plane was considered to generate each plot.

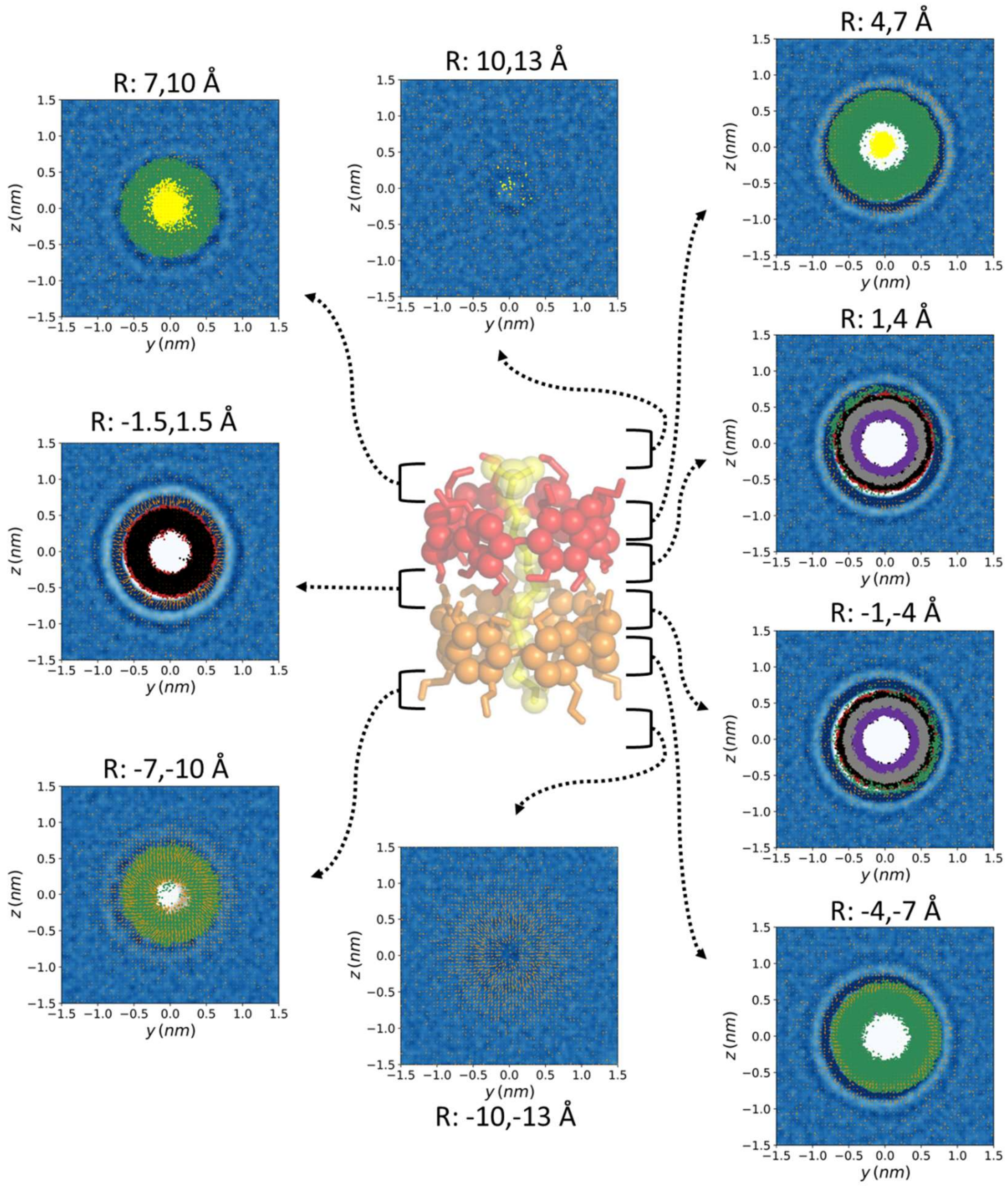

Figure S7.- Water density (blue-white gradient from highest to lowest) and average water dipole moment (orange vectors) around the **α-CD_2SDS_1 structure in aqueous solution** for a trajectory generated using the **AMBER BB-AM1 method**. Nine 3 Å width slices perpendicular to the symmetry axis of the complex were considered. The position of each slice is indicated by the brackets and arrows. The O2 (red), O3 (black), O4 (purple), O5 (grey), and O6 (green) atoms of the two cyclodextrins consisting the complex, together with the sulfur atom of the SDS molecule (yellow), are also represented. The last 10000 frames of one trajectory with the complex aligned to the O4 atoms were employed for these calculations. A resolution of 0.5x0.5 $Å^2$ in the YZ plane was considered to generate each plot.

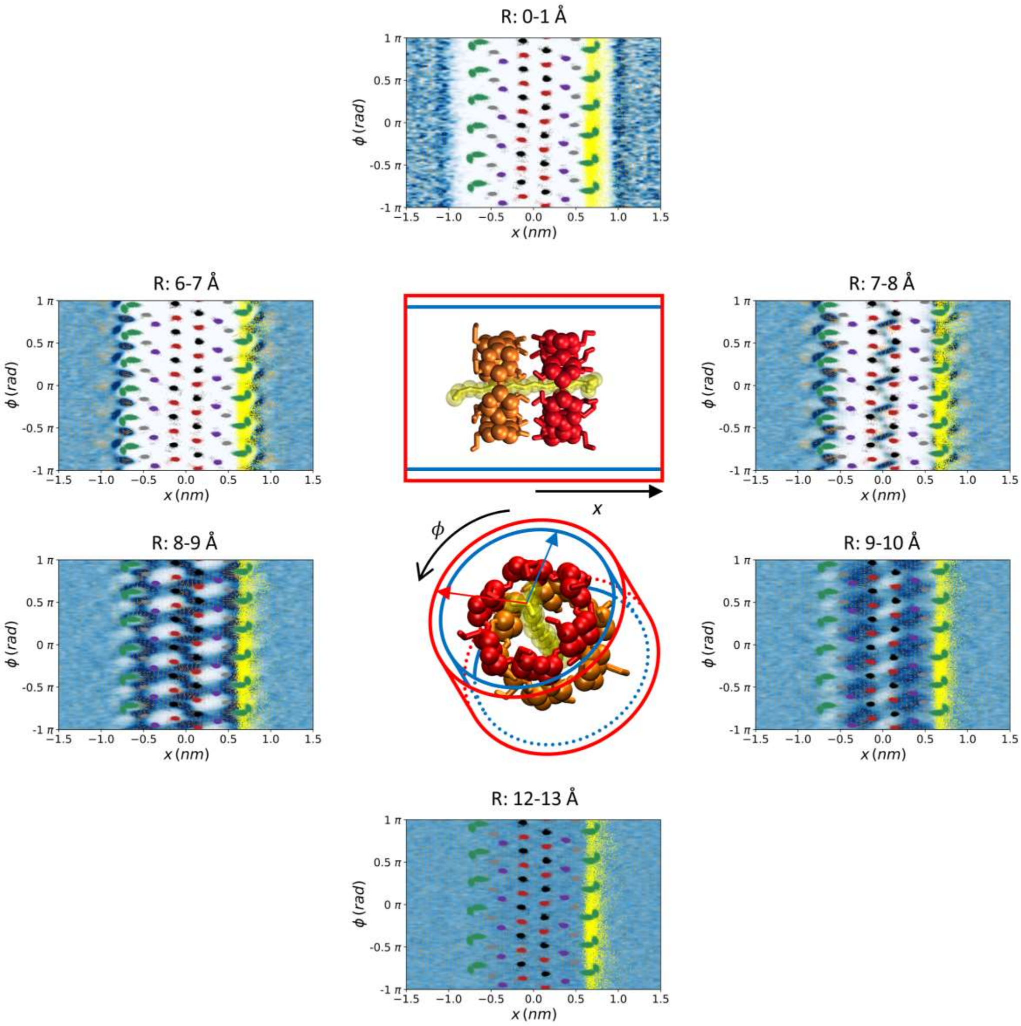

Figure S8.- Water density (blue-white gradient from highest to lowest) and average water dipole moment (orange vectors) around the **α-CD_2SDS_1 structure in aqueous solution** for a trajectory generated using the **HMR_2 method**. Thirteen 1 Å thick coaxial shells centered in the symmetry axis of the complex were considered but just the more representatives six are shown. Cylindrical coordinates are employed, for clarity. The distances to the symmetry axis corresponding to each shell is indicated in the figure. The O2 (red), O3 (black), O4 (purple), O5 (grey), and O6 (green) atoms of the two CDs consisting the complex, together with the sulfur atom of the SDS molecule (yellow), are also represented. The last 10000 frames of one trajectory with the complex aligned to the O4 atoms were employed for these calculations. A resolution of 0.1 rad in the ϕ axis and 0.5 Å in the X axis were considered to generate each plot.

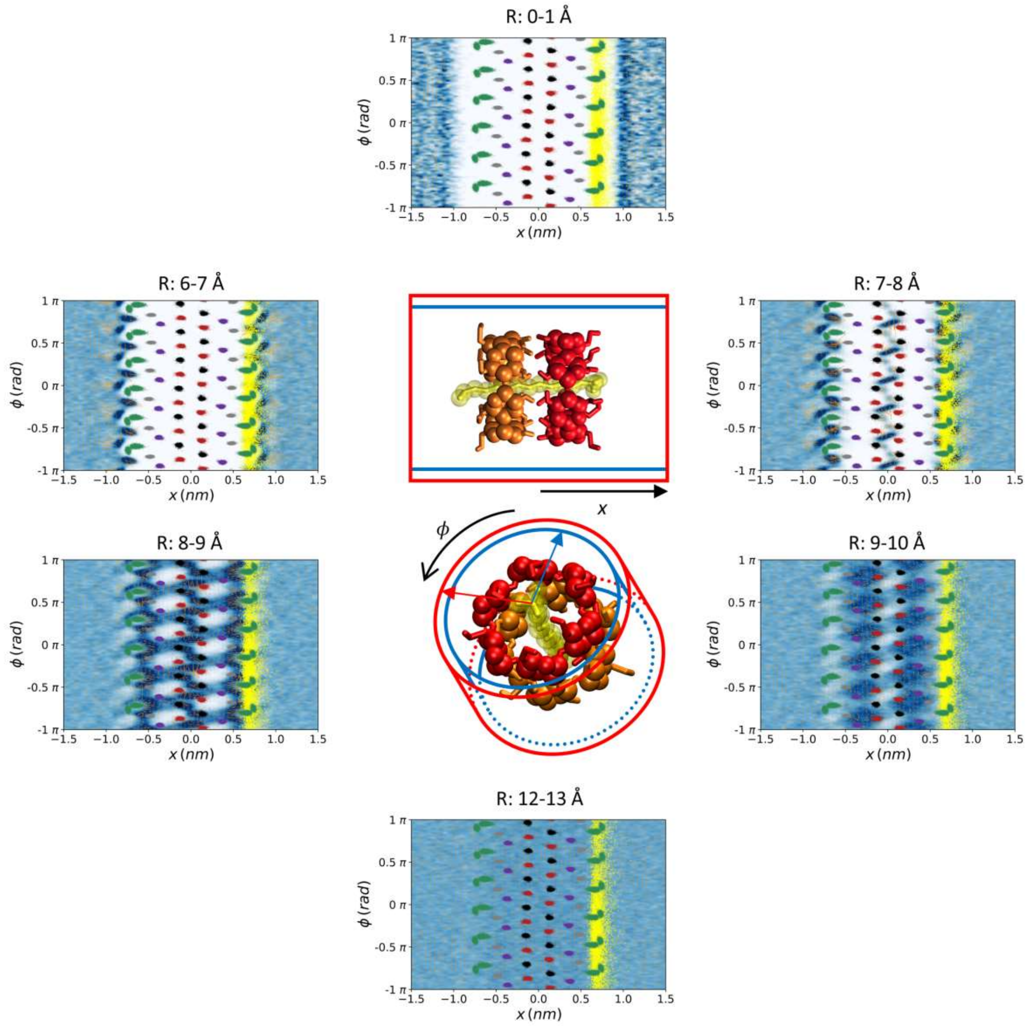

Figure S9.- Water density (blue-white gradient from highest to lowest) and average water dipole moment (orange vectors) around the **α-CD$_2$SDS$_1$ structure in aqueous solution** for a trajectory generated using the **HMR_7 method**. Thirteen 1 Å thick coaxial shells centered in the symmetry axis of the complex were considered but just the more representatives six are shown. Cylindrical coordinates are employed, for clarity. The distances to the symmetry axis corresponding to each shell is indicated in the figure. The O2 (red), O3 (black), O4 (purple), O5 (grey), and O6 (green) atoms of the two CDs consisting the complex, together with the sulfur atom of the SDS molecule (yellow), are also represented. The last 10000 frames of one trajectory with the complex aligned to the O4 atoms were employed for these calculations. A resolution of 0.1 rad in the ϕ axis and 0.5 Å in the X axis were considered to generate each plot.

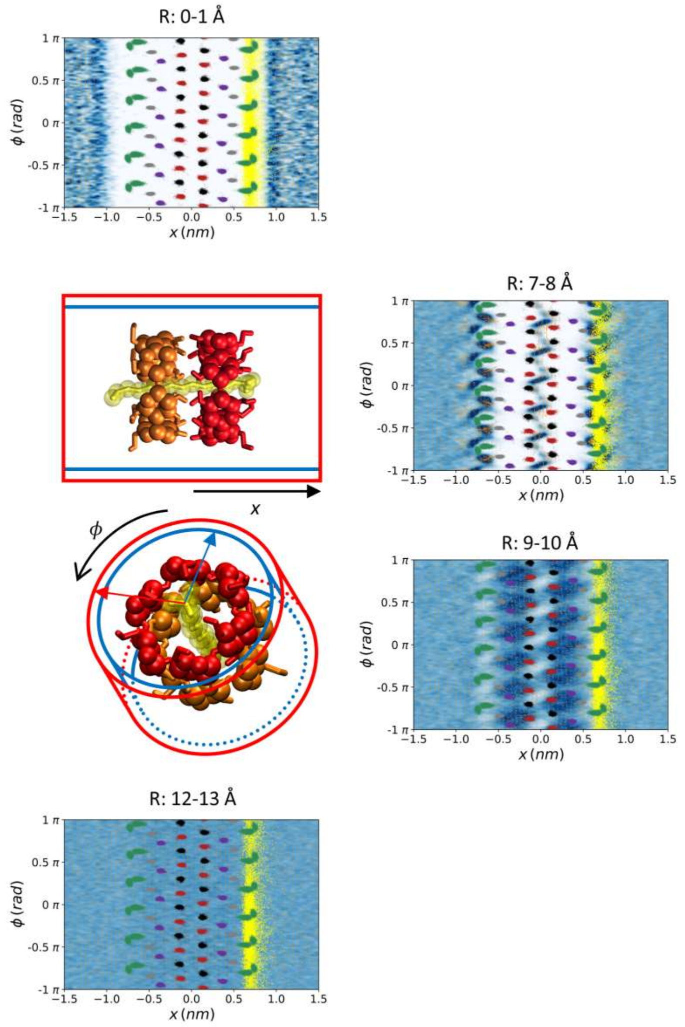

Figure S10.- Water density (blue-white gradient from highest to lowest) and average water dipole moment (orange vectors) around the **α-CD_2SDS_1 structure in aqueous solution** for a trajectory generated using the **H2Q_2 method**. Thirteen 1 Å thick coaxial shells centered in the symmetry axis of the complex were considered but just the more representatives six are shown. Cylindrical coordinates are employed, for clarity. The distances to the symmetry axis corresponding to each shell is indicated in the figure. The O2 (red), O3 (black), O4 (purple), O5 (grey), and O6 (green) atoms of the two CDs consisting the complex, together with the sulfur atom of the SDS molecule (yellow), are also represented. The last 10000 frames of one trajectory with the complex aligned to the O4 atoms were employed for these calculations. A resolution of 0.1 rad in the ϕ axis and 0.5 Å in the X axis were considered to generate each plot.

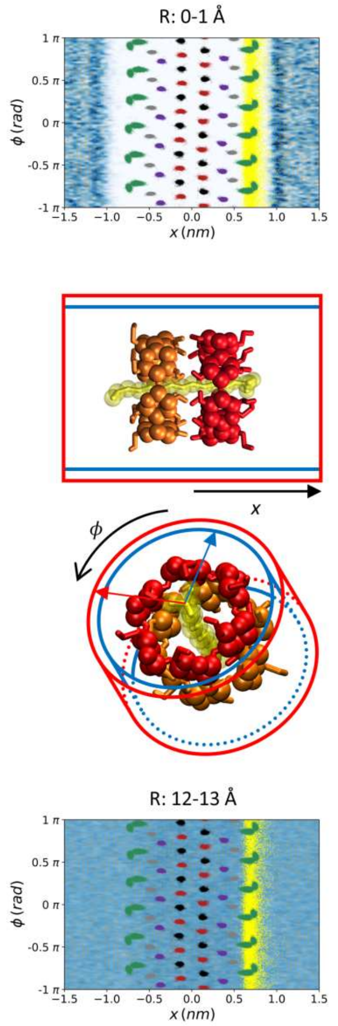

Figure S11.- Water density (blue-white gradient from highest to lowest) and average water dipole moment (orange vectors) around the **α-CD_2SDS_1 structure in aqueous solution** for a trajectory generated using the **H2Q_7 method**. Thirteen 1 Å thick coaxial shells centered in the symmetry axis of the complex were considered but just the more representatives six are shown. Cylindrical coordinates are employed, for clarity. The distances to the symmetry axis corresponding to each shell is indicated in the figure. The O2 (red), O3 (black), O4 (purple), O5 (grey), and O6 (green) atoms of the two CDs consisting the complex, together with the sulfur atom of the SDS molecule (yellow), are also represented. The last 10000 frames of one trajectory with the complex aligned to the O4 atoms were employed for these calculations. A resolution of 0.1 rad in the ϕ axis and 0.5 Å in the X axis were considered to generate each plot.

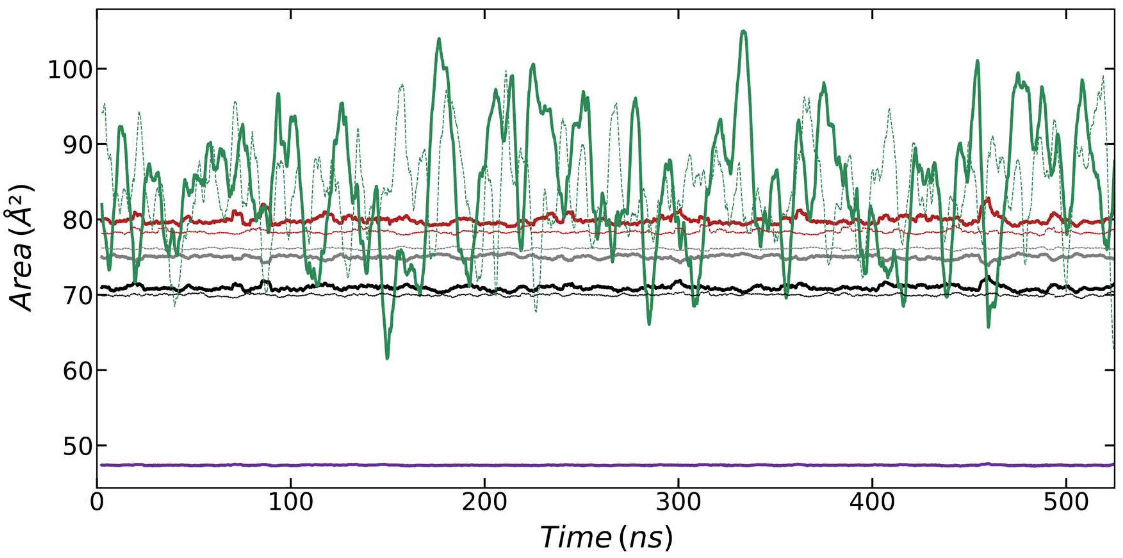

Figure S12.- Area of the hexagons formed by the O2 (red), O3 (black), O4 (purple), O5 (grey) and O6 (green) atoms of the CD molecules that are closer (solid lines) and further (dashed lines) from the SDS head. This plot corresponds to the simulation of a **α-CD_2SDS_1 structure in aqueous solution** for a trajectory generated using the **HMR_2 method.**

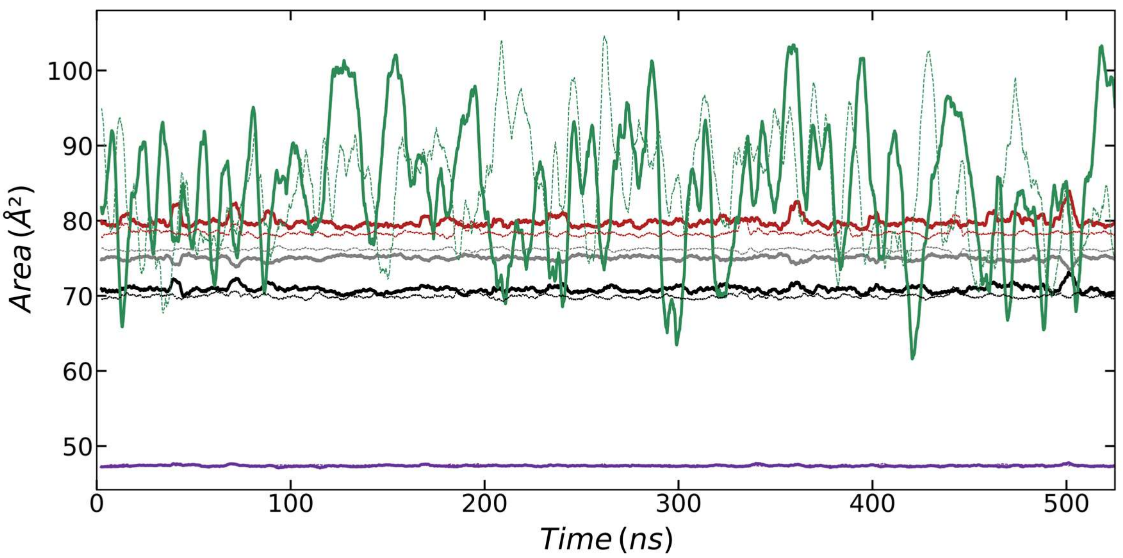

Figure S13.- Area of the hexagons formed by the O2 (red), O3 (black), O4 (purple), O5 (grey) and O6 (green) atoms of the CD molecules that are closer (solid lines) and further (dashed lines) from the SDS head. This plot corresponds to the simulation of a **α-CD_2SDS_1 structure in aqueous solution** for a trajectory generated using the **HMR_7 method.**

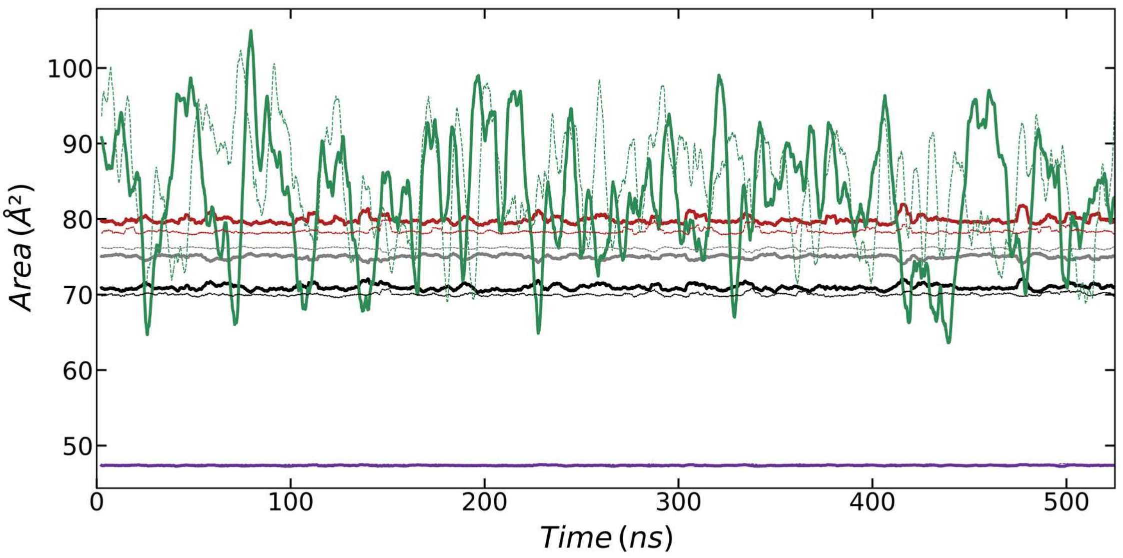

Figure S14.- Area of the hexagons formed by the O2 (red), O3 (black), O4 (purple), O5 (grey) and O6 (green) atoms of the CD molecules that are closer (solid lines) and further (dashed lines) from the SDS head. This plot corresponds to the simulation of a **α-CD_2SDS_1 structure in aqueous solution** for a trajectory generated using the **H2Q _2 method.**

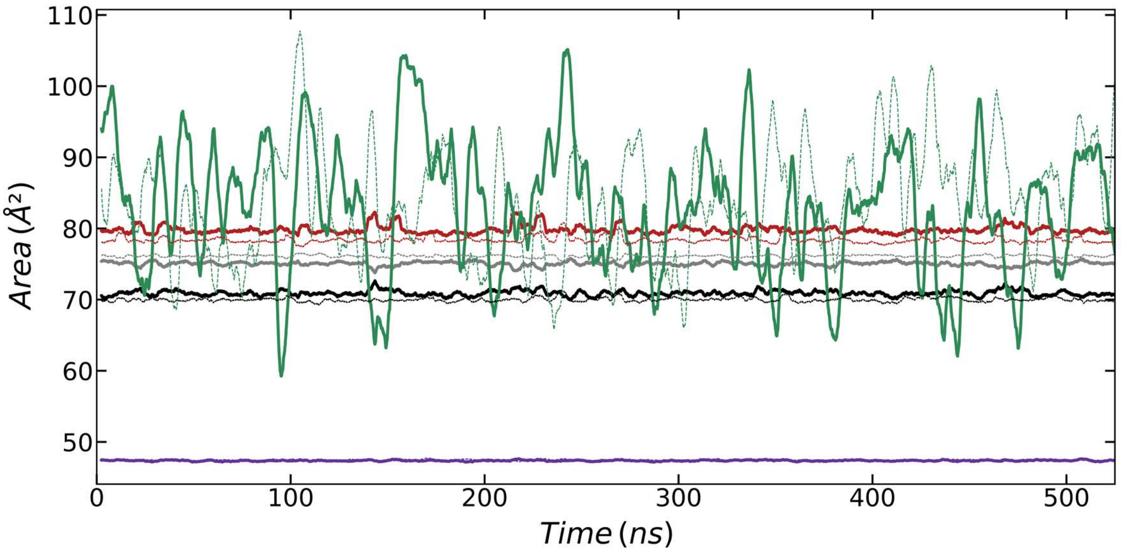

Figure S15.- Area of the hexagons formed by the O2 (red), O3 (black), O4 (purple), O5 (grey) and O6 (green) atoms of the CD molecules that are closer (solid lines) and further (dashed lines) from the SDS head. This plot corresponds to the simulation of a **α-CD_2SDS_1 structure in aqueous solution** for a trajectory generated using the **H2Q _7 method.**

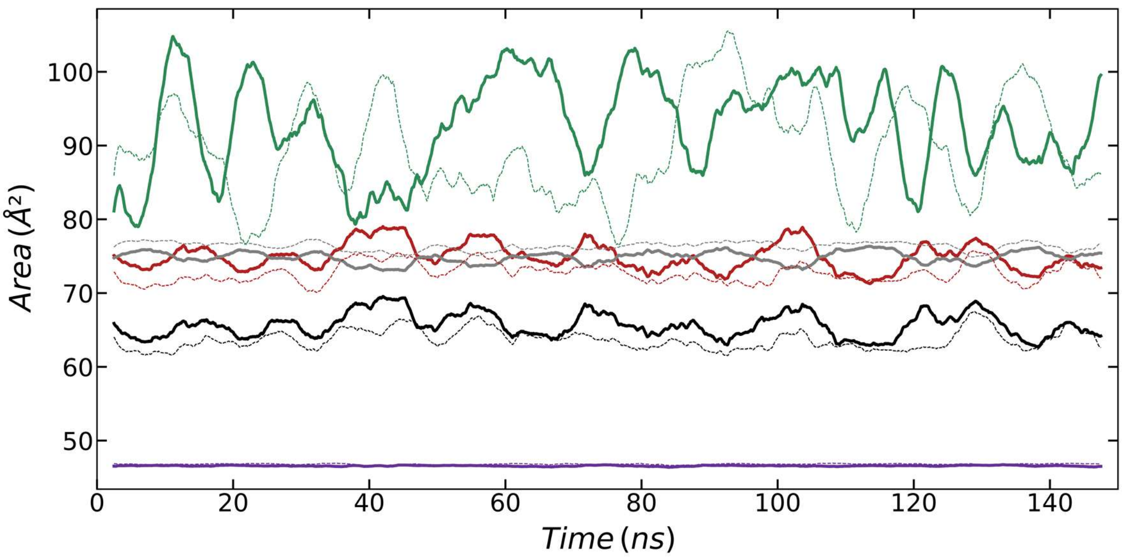

Figure S16.- Area of the hexagons formed by the O2 (red), O3 (black), O4 (purple), O5 (grey) and O6 (green) atoms of the CD molecules that are closer (solid lines) and further (dashed lines) from the SDS head. This plot corresponds to the simulation of a **α-CD_2SDS_1 structure in aqueous solution** for a trajectory generated using the **AMBER force field with RESP charges.**

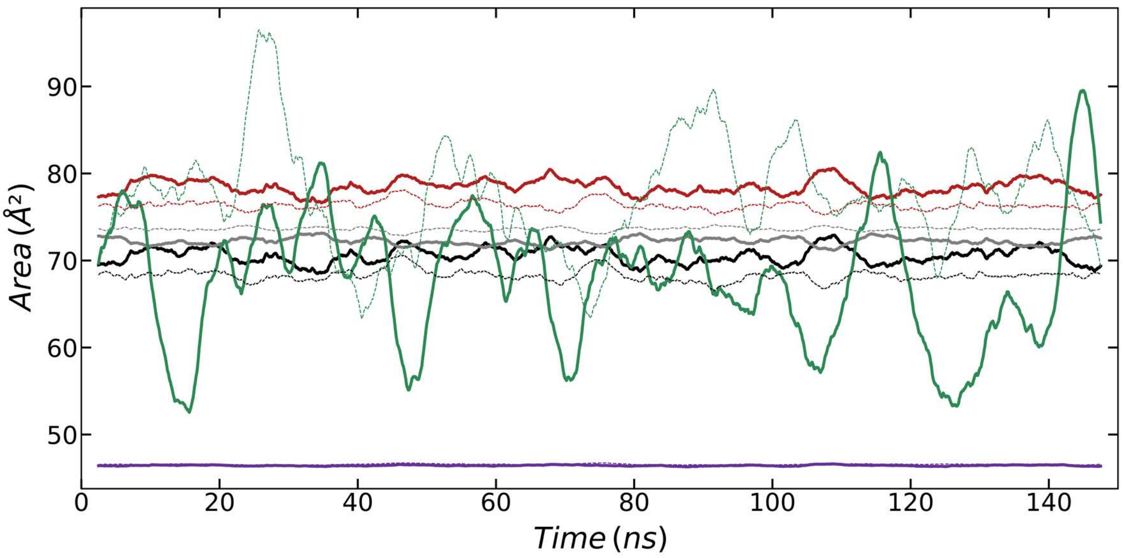

Figure S17.- Area of the hexagons formed by the O2 (red), O3 (black), O4 (purple), O5 (grey) and O6 (green) atoms of the CD molecules that are closer (solid lines) and further (dashed lines) from the SDS head. This plot corresponds to the simulation of a **α-CD_2SDS_1 structure in aqueous solution** for a trajectory generated using the **AMBER force field with AM1-BCC charges.**

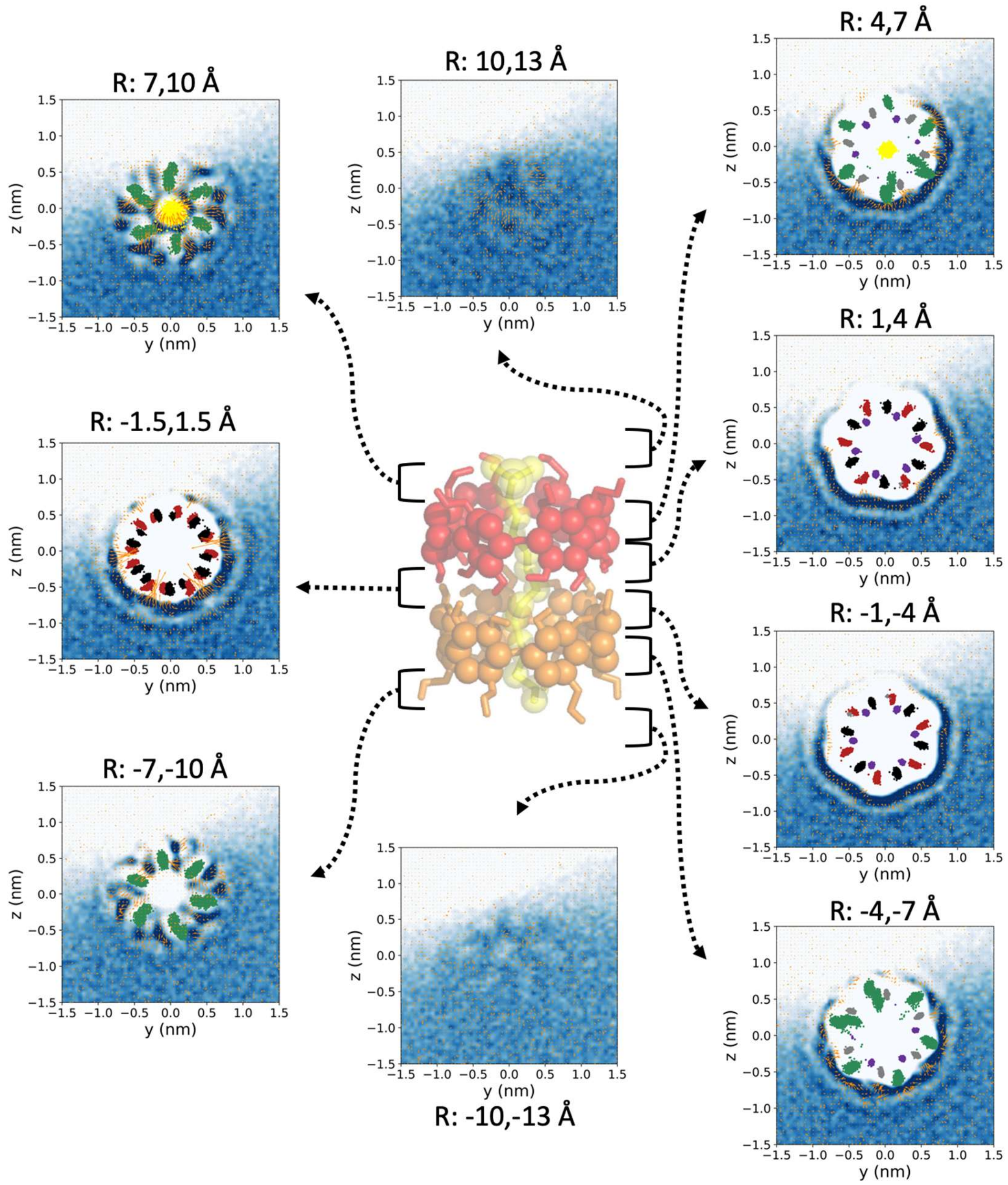

Figure S18.- Water density (blue-white gradient from highest to lowest) and average water dipole moment (orange vectors) around the **α-CD$_2$SDS$_1$ structure at the water/air interface** for a trajectory generated using the **HMR_2 method**. Nine 3 Å width slices perpendicular to the symmetry axis of the complex were considered. The position of each slice is indicated by the brackets and arrows. The O2 (red), O3 (black), O4 (purple), O5 (grey), and O6 (green) atoms of the two CDs consisting the complex, together with the sulfur atom of the SDS molecule (yellow), are also represented. A selection of the last 10000 frames of one trajectory with the complex aligned to the O4 atoms, corresponding to the frames where **the GPU number 1 of the first CD has the minimum hydration**, were employed for these calculations. A resolution of 0.1 rad in the ϕ axis and 0.5 Å in the X axis were considered to generate each plot.

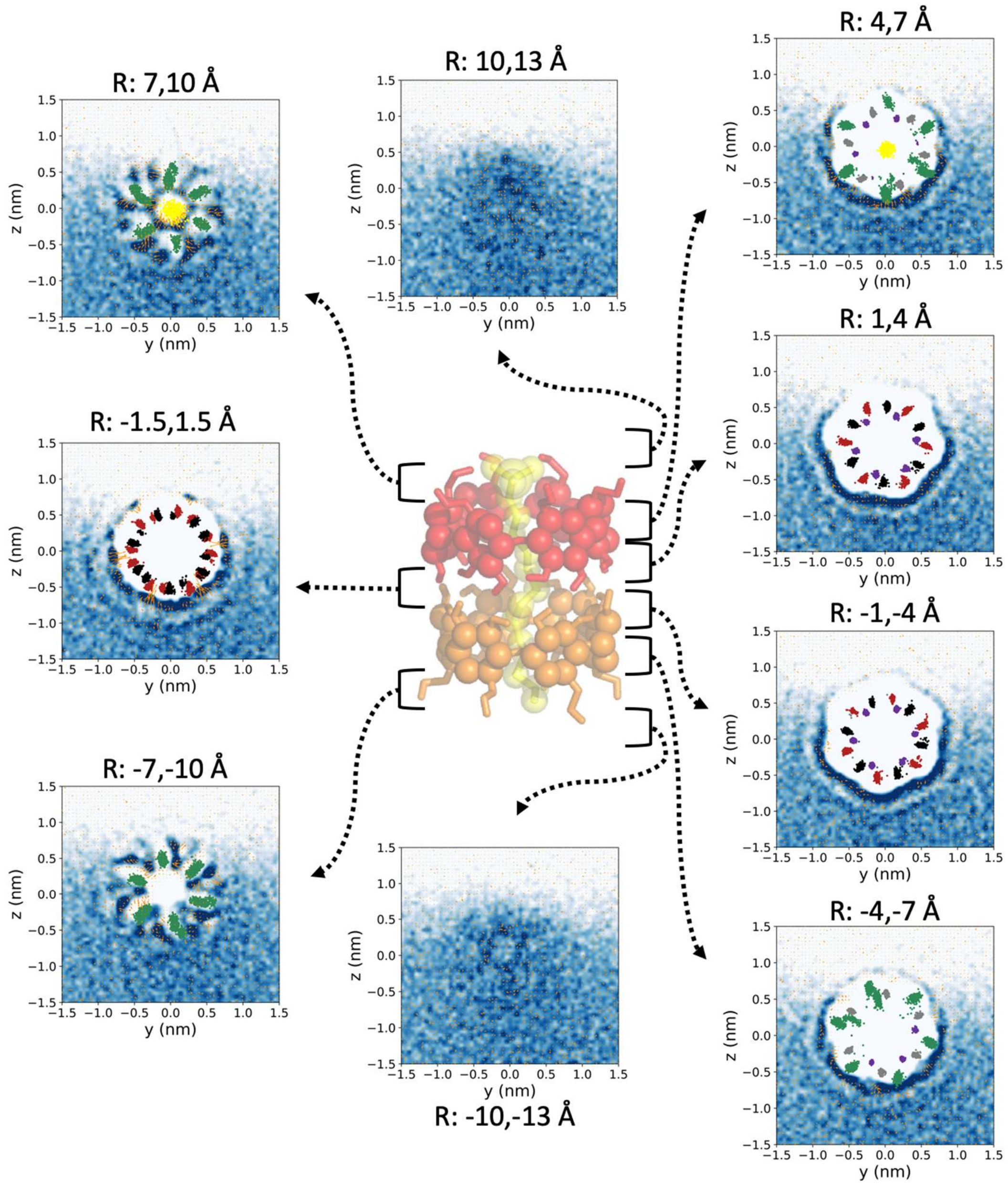

Figure S19.- Water density (blue-white gradient from highest to lowest) and average water dipole moment (orange vectors) around the **α-CD_2SDS_1 structure at the water/air interface** for a trajectory generated using the **HMR_7 method**. Nine 3 Å width slices perpendicular to the symmetry axis of the complex were considered. The position of each slice is indicated by the brackets and arrows. The O2 (red), O3 (black), O4 (purple), O5 (grey), and O6 (green) atoms of the two CDs consisting the complex, together with the sulfur atom of the SDS molecule (yellow), are also represented. A selection of the last 10000 frames of one trajectory with the complex aligned to the O4 atoms, corresponding to the frames where **the GPU number 1 of the first CD has the minimum hydration**, were employed for these calculations. A resolution of 0.1 rad in the ϕ axis and 0.5 Å in the X axis were considered to generate each plot.

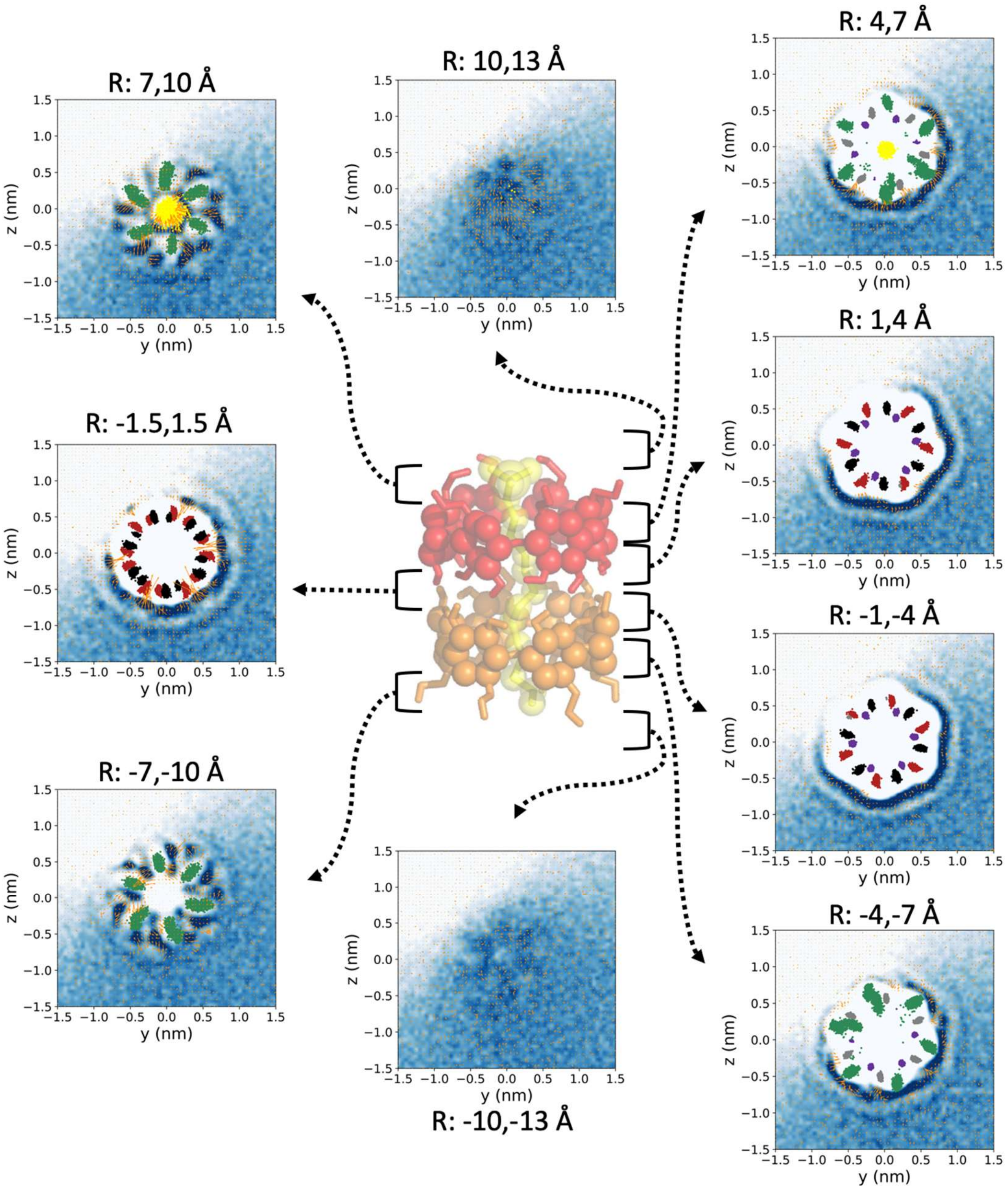

Figure S20.- Water density (blue-white gradient from highest to lowest) and average water dipole moment (orange vectors) around the **α-CD_2SDS_1 structure at the water/air interface** for a trajectory generated using the **H2Q_2 method**. Nine 3 Å width slices perpendicular to the symmetry axis of the complex were considered. The position of each slice is indicated by the brackets and arrows. The O2 (red), O3 (black), O4 (purple), O5 (grey), and O6 (green) atoms of the two CDs consisting the complex, together with the sulfur atom of the SDS molecule (yellow), are also represented. A selection of the last 10000 frames of one trajectory with the complex aligned to the O4 atoms, corresponding to the frames where **the GPU number 1 of the first CD has the minimum hydration**, were employed for these calculations. A resolution of 0.1 rad in the ϕ axis and 0.5 Å in the X axis were considered to generate each plot.

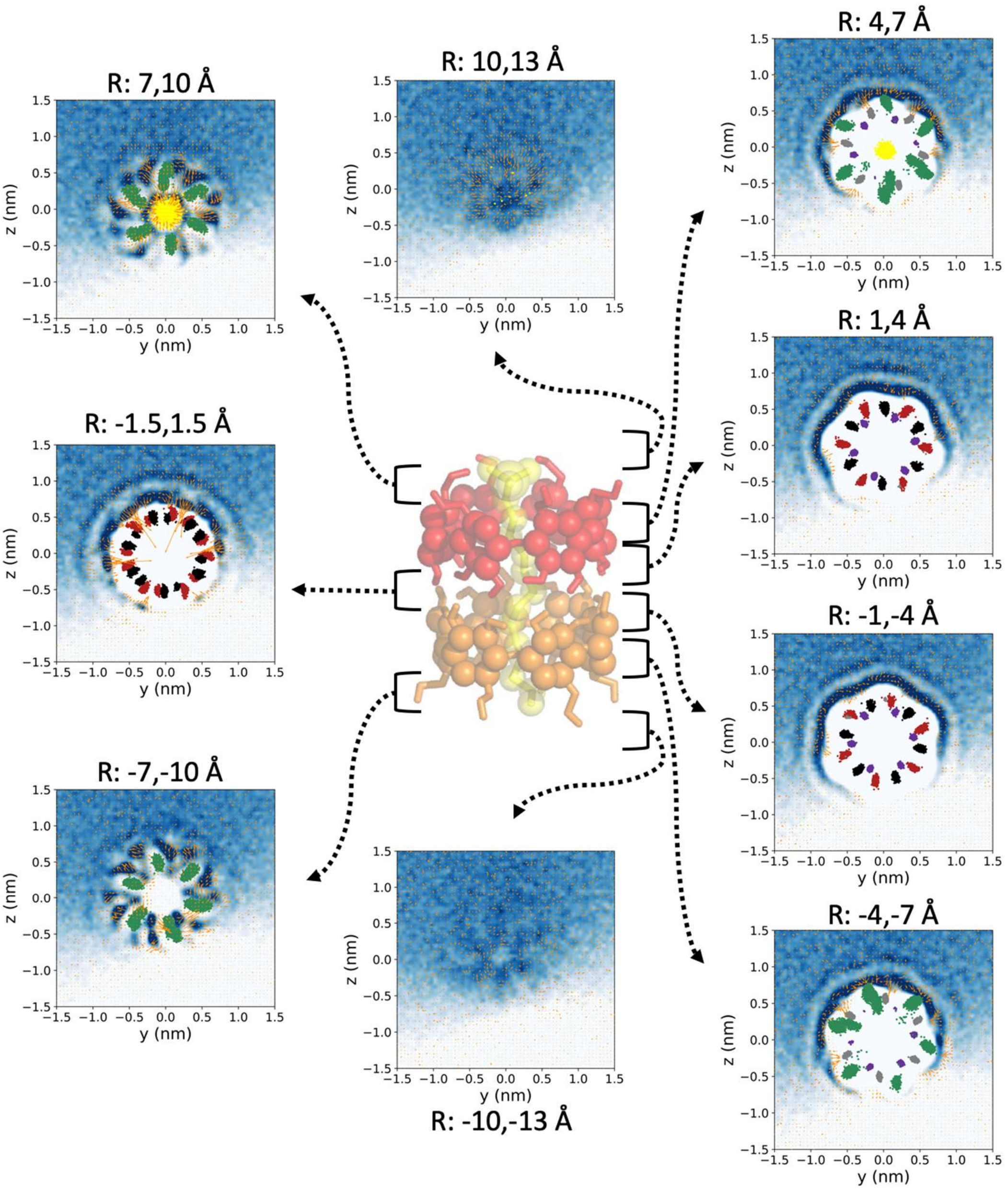

Figure S21.- Water density (blue-white gradient from highest to lowest) and average water dipole moment (orange vectors) around the **α-CD_2SDS_1 structure at the water/air interface** for a trajectory generated using the **H2Q_7 method**. Nine 3 Å width slices perpendicular to the symmetry axis of the complex were considered. The position of each slice is indicated by the brackets and arrows. The O2 (red), O3 (black), O4 (purple), O5 (grey), and O6 (green) atoms of the two CDs consisting the complex, together with the sulfur atom of the SDS molecule (yellow), are also represented. A selection of the last 10000 frames of one trajectory with the complex aligned to the O4 atoms, corresponding to the frames where **the GPU number 1 of the first CD has the minimum hydration**, were employed for these calculations. A resolution of 0.1 rad in the ϕ axis and 0.5 Å in the X axis were considered to generate each plot.

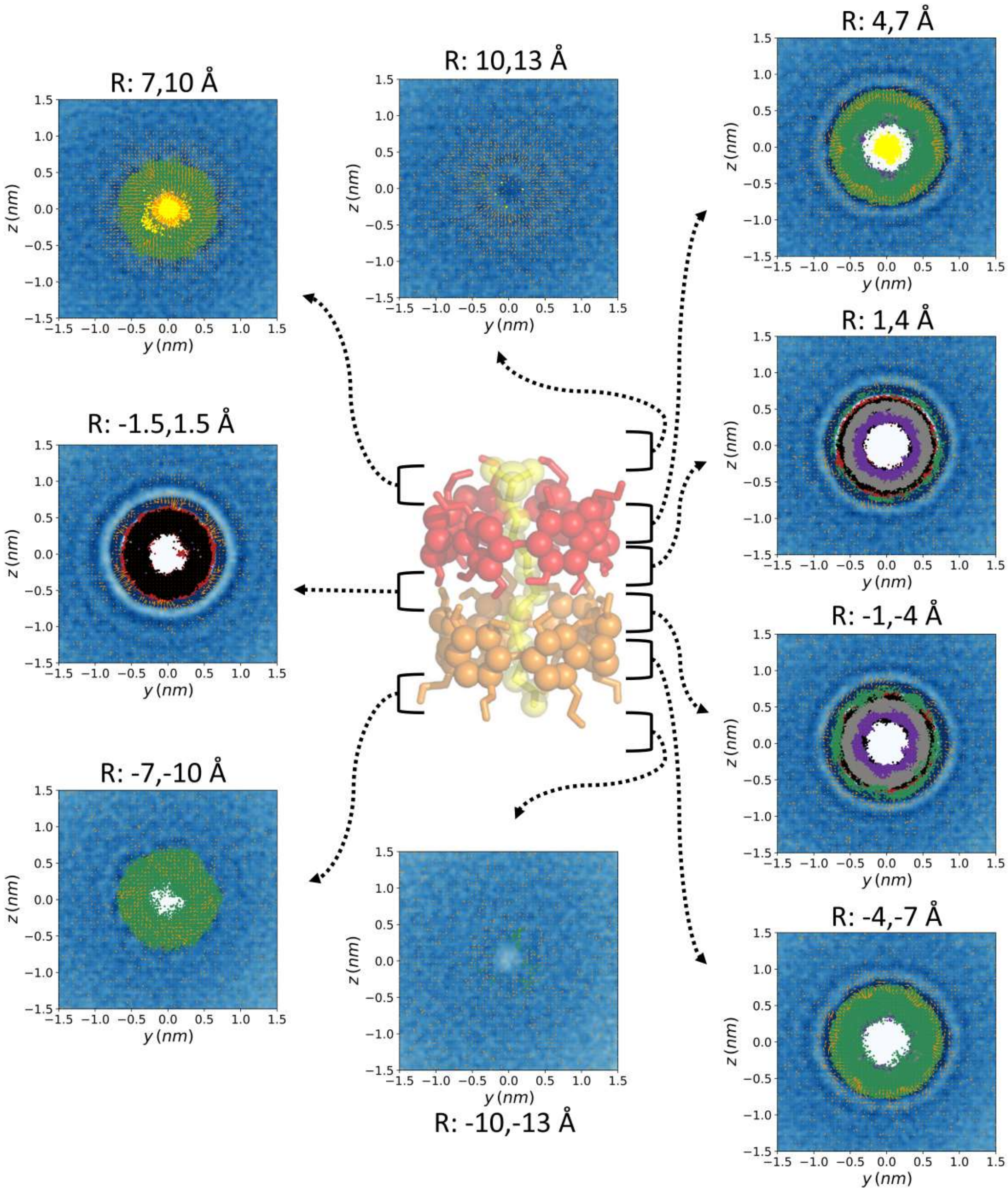

Figure S22.- Water density (blue-white gradient from highest to lowest) and average water dipole moment (orange vectors) around the **α-CD_2SDS_1 structure at the water/air interface** for a trajectory generated using the **AMBER BB-RESP method**. Nine 3 Å width slices perpendicular to the symmetry axis of the complex were considered. The position of each slice is indicated by the brackets and arrows. The O2 (red), O3 (black), O4 (purple), O5 (grey), and O6 (green) atoms of the two CDs consisting the complex, together with the sulfur atom of the SDS molecule (yellow), are also represented. The last 10000 frames of one trajectory with the complex aligned to the O4 atoms were employed for these calculations. A resolution of 0.5x0.5 $Å^2$ in the YZ plane was considered to generate each plot.

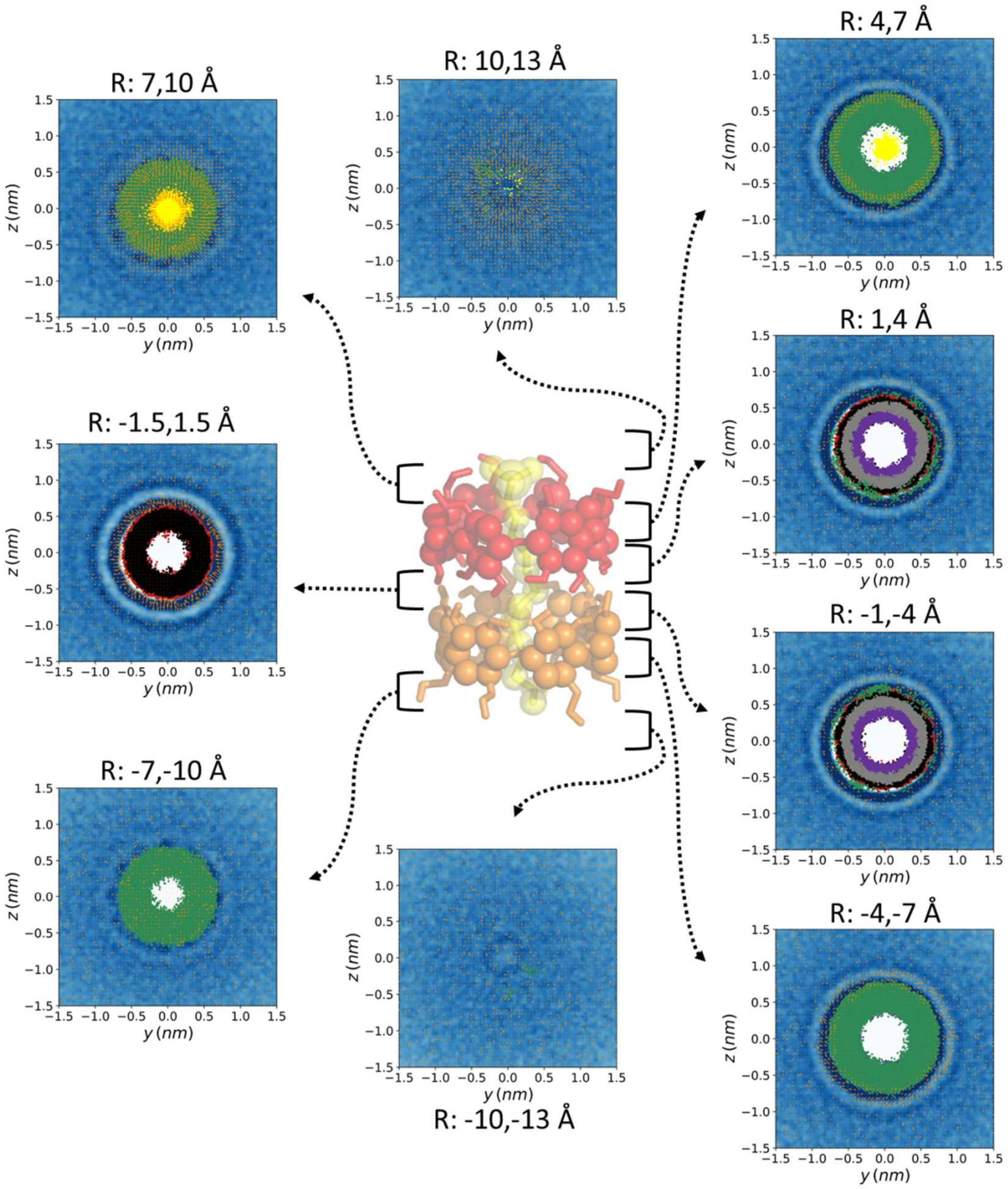

Figure S23.- Water density (blue-white gradient from highest to lowest) and average water dipole moment (orange vectors) around the **α-CD_2SDS_1 structure at the water/air interface** for a trajectory generated using the **AMBER BB-AM1 method**. Nine 3 Å width slices perpendicular to the symmetry axis of the complex were considered. The position of each slice is indicated by the brackets and arrows. The O2 (red), O3 (black), O4 (purple), O5 (grey), and O6 (green) atoms of the two CDs consisting the complex, together with the sulfur atom of the SDS molecule (yellow), are also represented. The last 10000 frames of one trajectory with the complex aligned to the O4 atoms were employed for these calculations. A resolution of 0.5x0.5 $Å^2$ in the YZ plane was considered to generate each plot.

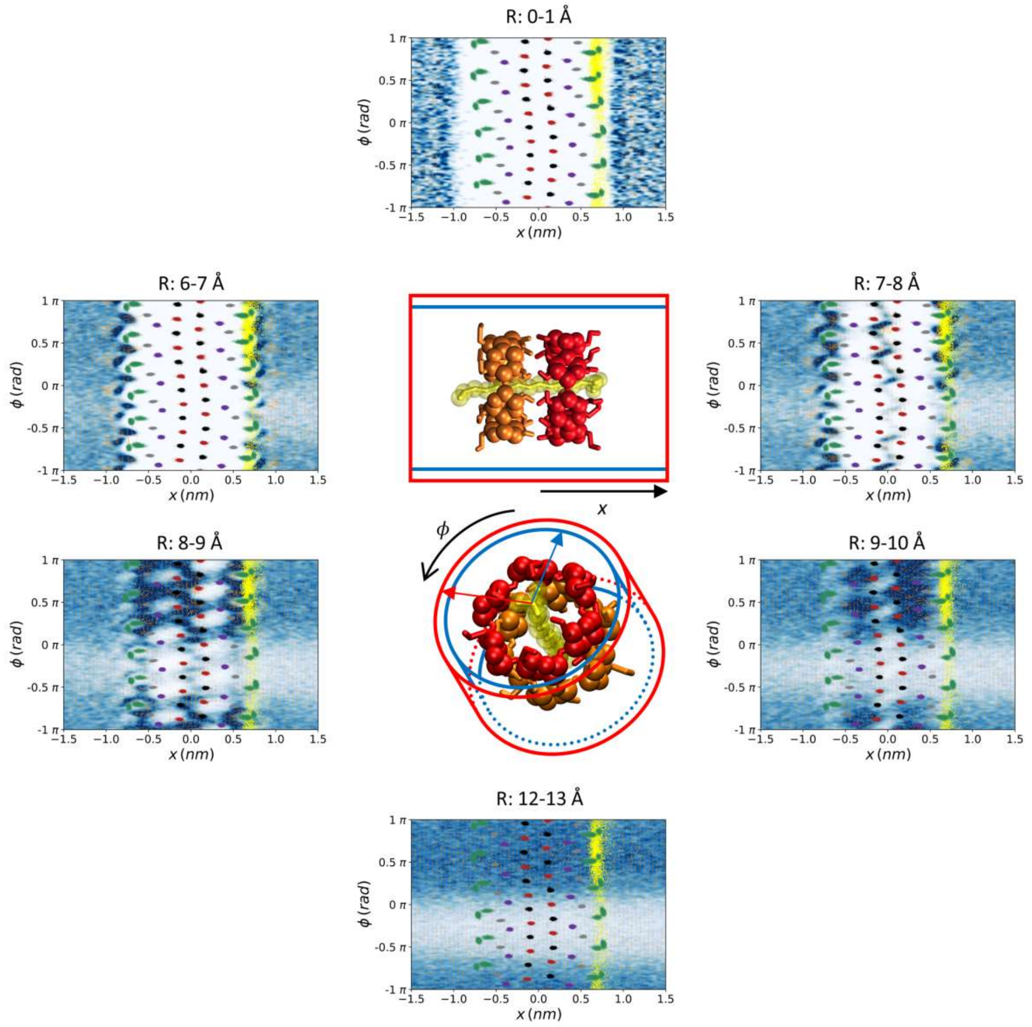

Figure S24.- Water density (blue-white gradient from highest to lowest) and average water dipole moment (orange vectors) around the **α-CD_2SDS_1 structure at the water/air interface** for a trajectory generated using the **HMR_2 method**. Nine 3 Å width slices perpendicular to the symmetry axis of the complex were considered. The position of each slice is indicated by the brackets and arrows. The O2 (red), O3 (black), O4 (purple), O5 (grey), and O6 (green) atoms of the two CDs consisting the complex, together with the sulfur atom of the SDS molecule (yellow), are also represented. A selection of the last 10000 frames of one trajectory with the complex aligned to the O4 atoms, corresponding to the frames where **the GPU number 1 of the first CD has the minimum hydration**, were employed for these calculations. A resolution of 0.5x0.5 $Å^2$ in the YZ plane was considered to generate each plot.

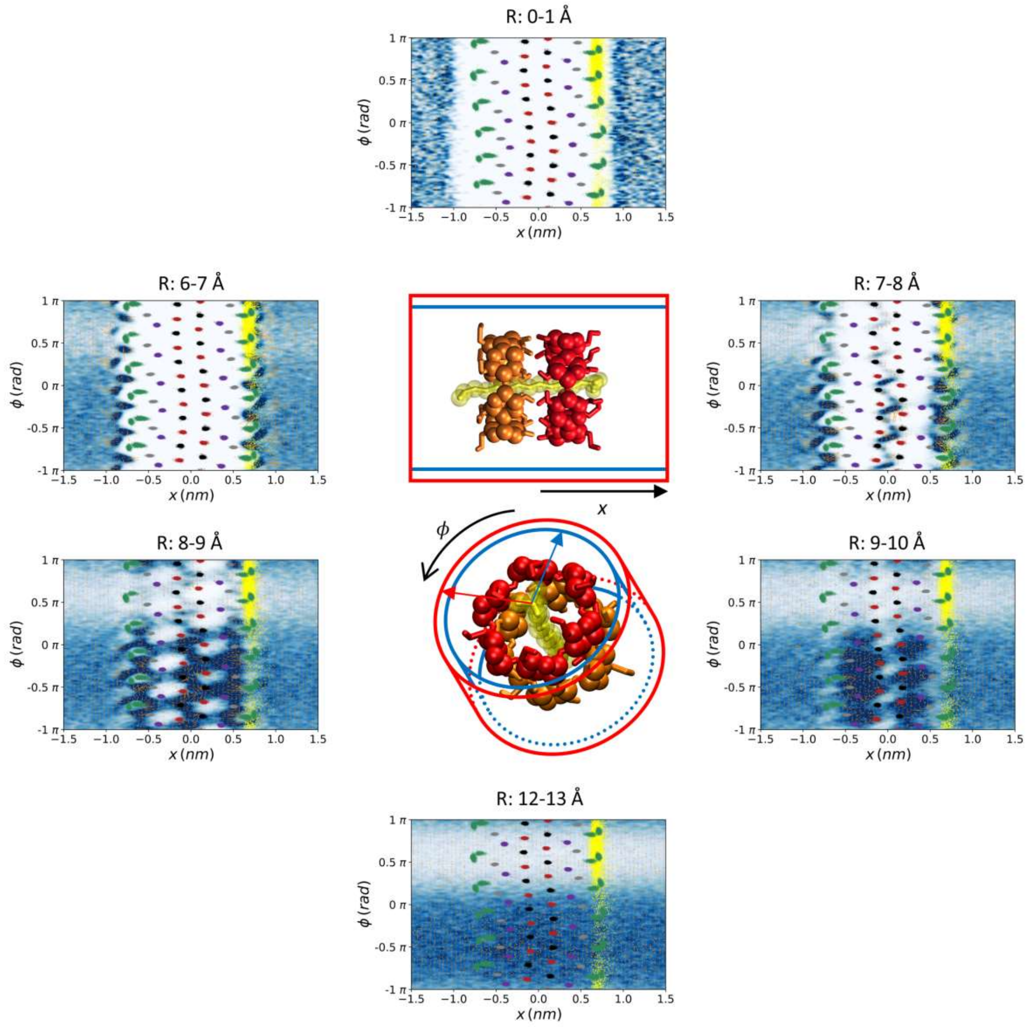

Figure S25.- Water density (blue-white gradient from highest to lowest) and average water dipole moment (orange vectors) around the **α-CD_2SDS_1 structure at the water/air interface** for a trajectory generated using the **HMR_7 method**. Nine 3 Å width slices perpendicular to the symmetry axis of the complex were considered. The position of each slice is indicated by the brackets and arrows. The O2 (red), O3 (black), O4 (purple), O5 (grey), and O6 (green) atoms of the two CDs consisting the complex, together with the sulfur atom of the SDS molecule (yellow), are also represented. A selection of the last 10000 frames of one trajectory with the complex aligned to the O4 atoms, corresponding to the frames where **the GPU number 1 of the first CD has the minimum hydration**, were employed for these calculations. A resolution of 0.1 rad in the ϕ axis and 0.5 Å in the X axis were considered to generate each plot.

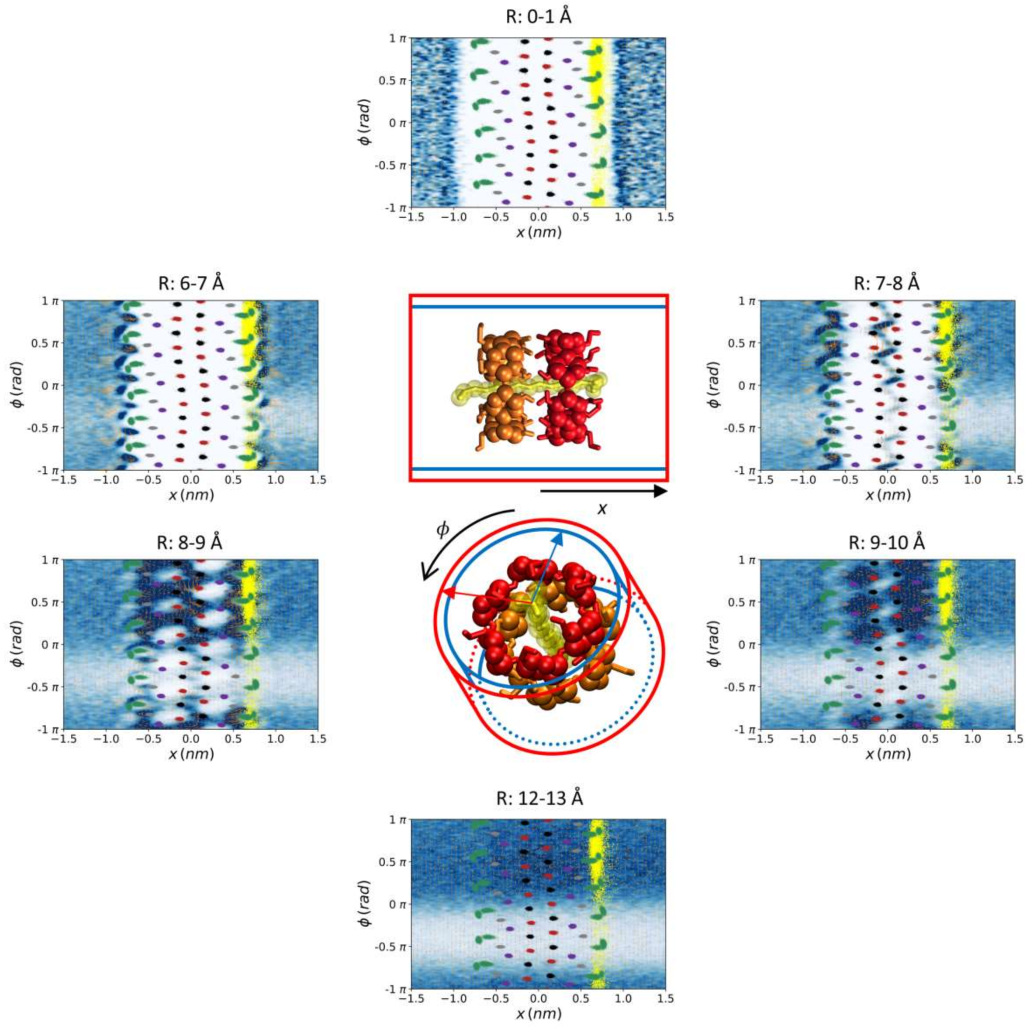

Figure S26.- Water density (blue-white gradient from highest to lowest) and average water dipole moment (orange vectors) around the **α-CD_2SDS_1 structure at the water/air interface** for a trajectory generated using the **H2Q_2 method**. Nine 3 Å width slices perpendicular to the symmetry axis of the complex were considered. The position of each slice is indicated by the brackets and arrows. The O2 (red), O3 (black), O4 (purple), O5 (grey), and O6 (green) atoms of the two CDs consisting the complex, together with the sulfur atom of the SDS molecule (yellow), are also represented. A selection of the last 10000 frames of one trajectory with the complex aligned to the O4 atoms, corresponding to the frames where **the GPU number 1 of the first CD has the minimum hydration**, were employed for these calculations. A resolutionof 0.1 rad in the ϕ axis and 0.5 Å in the X axis were considered to generate each plot.

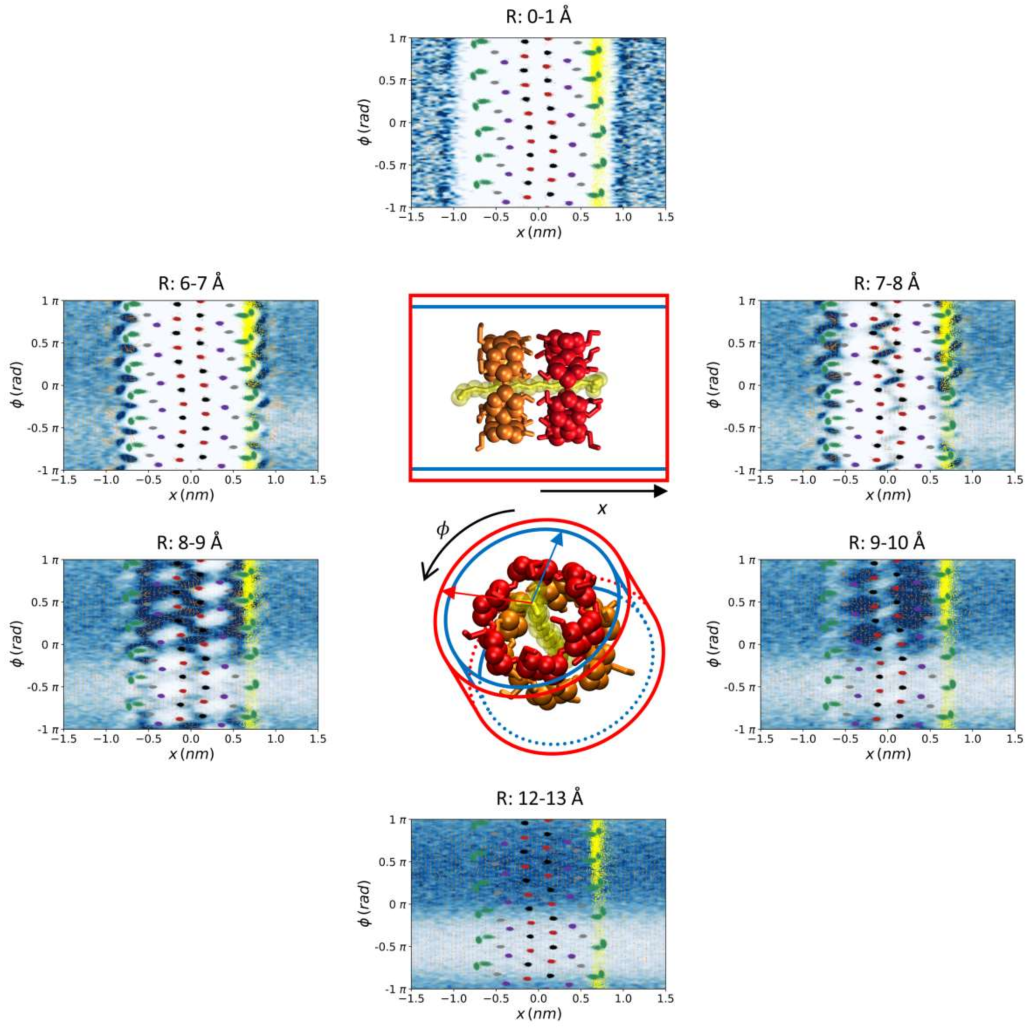

Figure S27.- Water density (blue-white gradient from highest to lowest) and average water dipole moment (orange vectors) around the **α-CD_2SDS_1 structure at the water/air interface** for a trajectory generated using the **H2Q_7 method**. Nine 3 Å width slices perpendicular to the symmetry axis of the complex were considered. The position of each slice is indicated by the brackets and arrows. The O2 (red), O3 (black), O4 (purple), O5 (grey), and O6 (green) atoms of the two CDs consisting the complex, together with the sulfur atom of the SDS molecule (yellow), are also represented. A selection of the last 10000 frames of one trajectory with the complex aligned to the O4 atoms, corresponding to the frames where **the GPU number 1 of the first CD has the minimum hydration**, were employed for these calculations. A resolution of 0.1 rad in the ϕ axis and 0.5 Å in the X axis were considered to generate each plot.

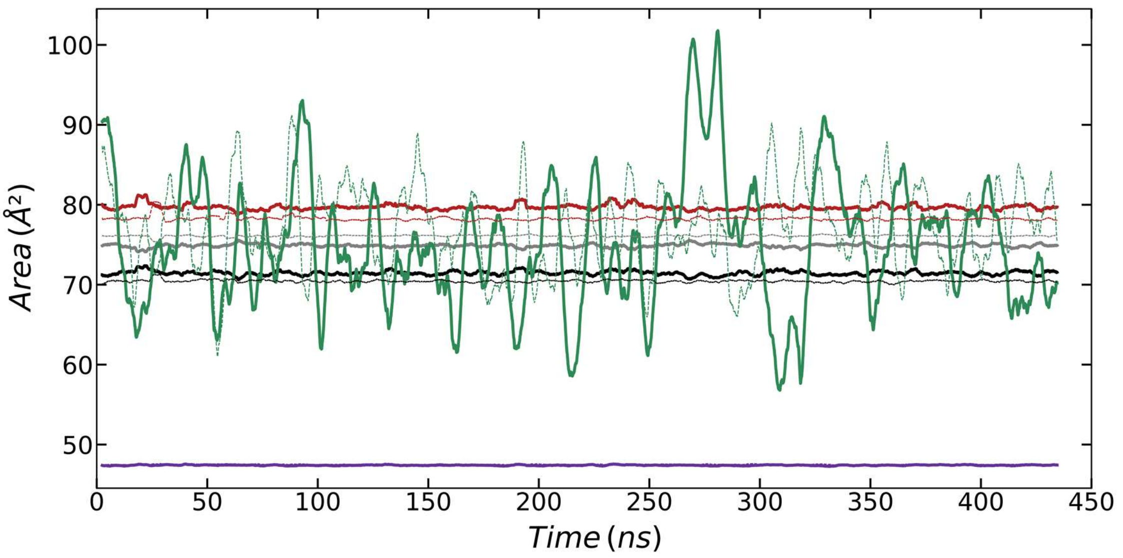

Figure S28.- Area of the hexagons formed by the O2 (red), O3 (black), O4 (purple), O5 (grey) and O6 (green) atoms of the CD molecules that are closer (solid lines) and further (dashed lines) from the SDS head. This plot corresponds to the simulation of the **α-CD_2SDS_1 structure at the water/air interface** for a trajectory generated using the **HMR_2 method.**

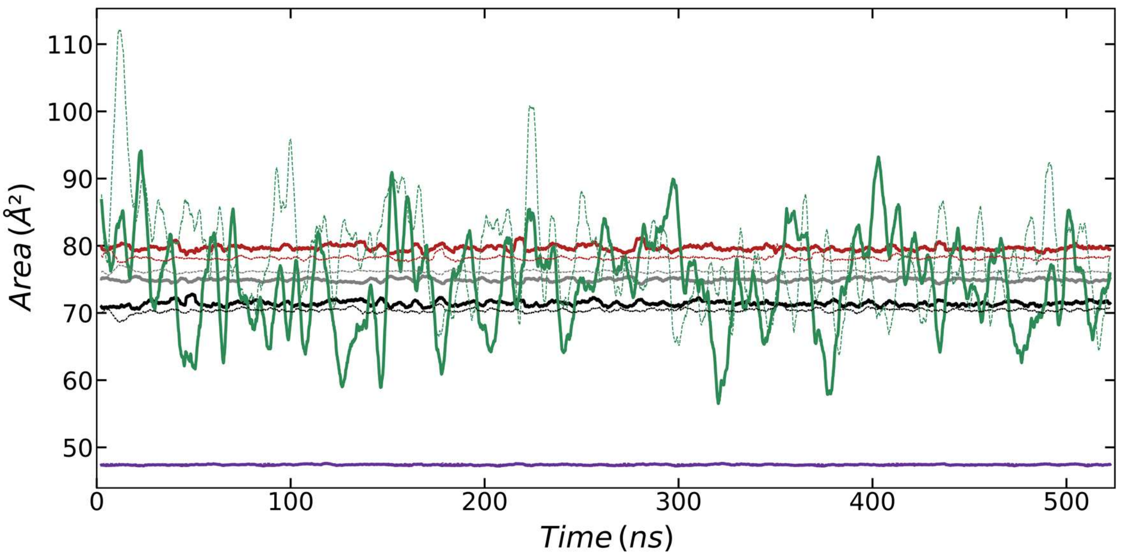

Figure S29.- Area of the hexagons formed by the O2 (red), O3 (black), O4 (purple), O5 (grey) and O6 (green) atoms of the CD molecules that are closer (solid lines) and further (dashed lines) from the SDS head. This plot corresponds to the simulation of the **α-CD_2SDS_1 structure at the water/air interface** for a trajectory generated using the **HMR_7 method.**

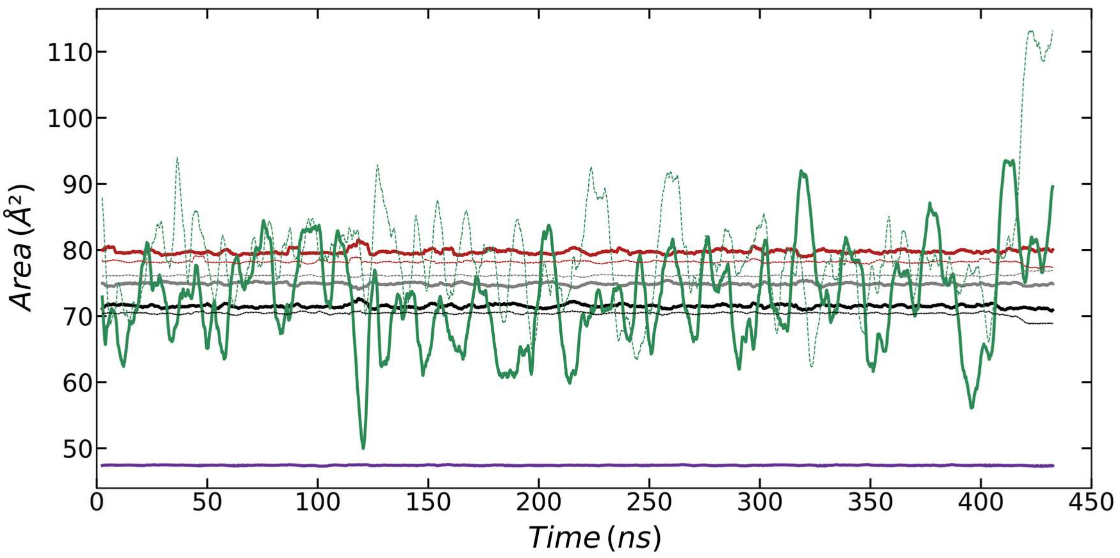

Figure S30.- Area of the hexagons formed by the O2 (red), O3 (black), O4 (purple), O5 (grey) and O6 (green) atoms of the CD molecules that are closer (solid lines) and further (dashed lines) from the SDS head. This plot corresponds to the simulation of the **α-CD_2SDS_1 structure at the water/air interface** for a trajectory generated using the **H2Q _2 method.**

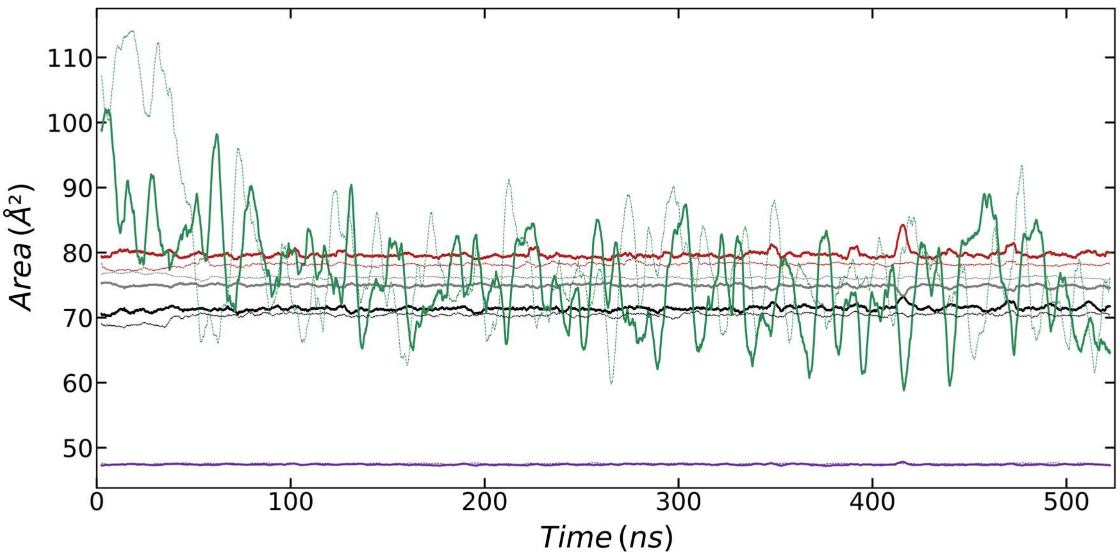

Figure S31.- Area of the hexagons formed by the O2 (red), O3 (black), O4 (purple), O5 (grey) and O6 (green) atoms of the CD molecules that are closer (solid lines) and further (dashed lines) from the SDS head. This plot corresponds to the simulation of the **α-CD_2SDS_1 structure at the water/air interface** for a trajectory generated using the **H2Q_7 method.**

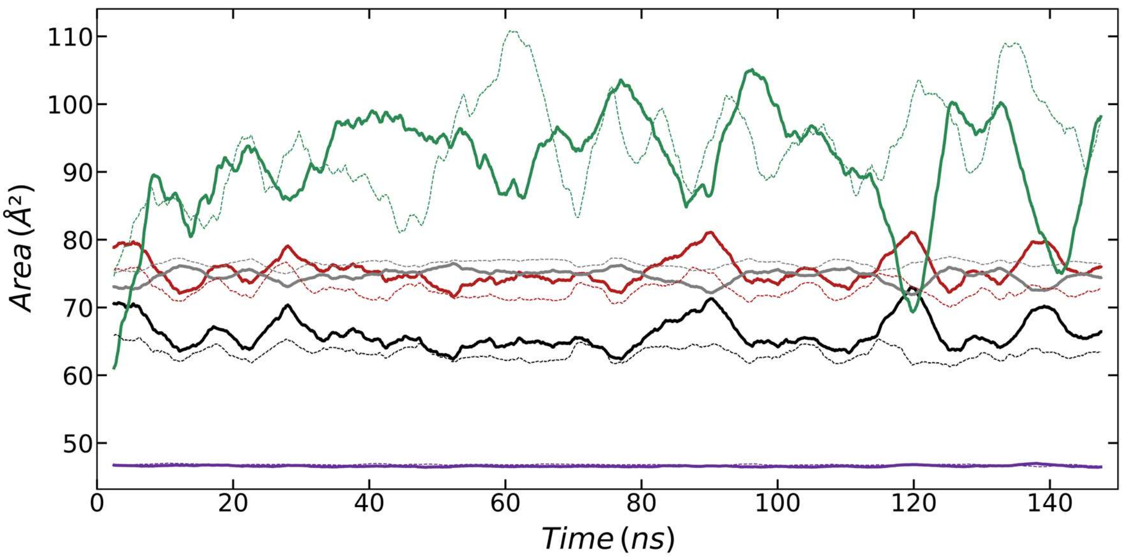

Figure S32.- Area of the hexagons formed by the O2 (red), O3 (black), O4 (purple), O5 (grey) and O6 (green) atoms of the CD molecules that are closer (solid lines) and further (dashed lines) from the SDS head. This plot corresponds to the simulation of the **α-CD_2SDS_1 structure at the water/air interface** for a trajectory generated using the **AMBER BB-RESP method.**

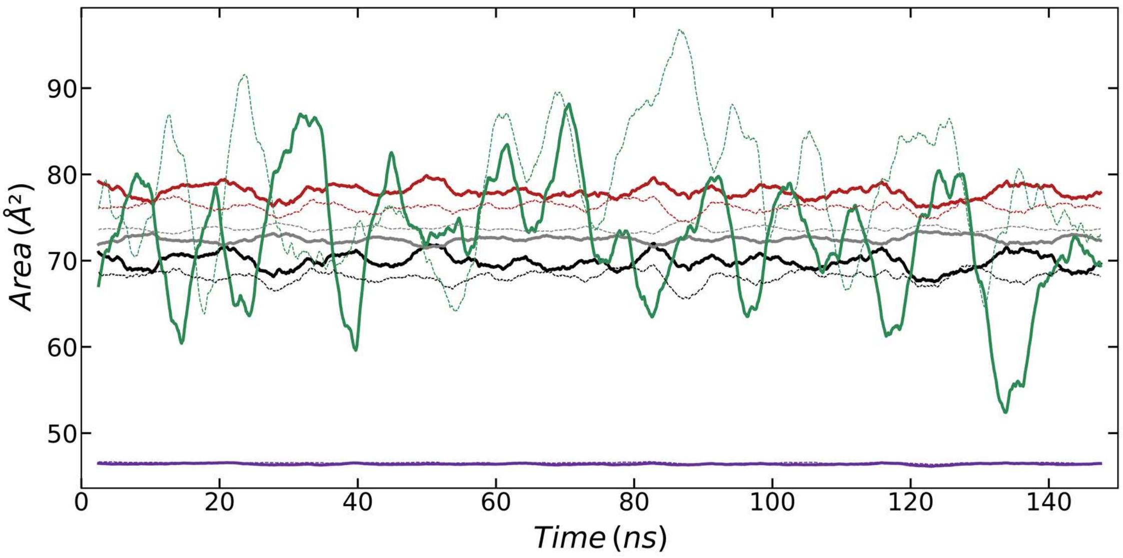

Figure S33.- Area of the hexagons formed by the O2 (red), O3 (black), O4 (purple), O5 (grey) and O6 (green) atoms of the CD molecules that are closer (solid lines) and further (dashed lines) from the SDS head. This plot corresponds to the simulation of the **α-CD_2SDS_1 structure at the water/air interface** for a trajectory generated using the **AMBER BB-AM1 method.**

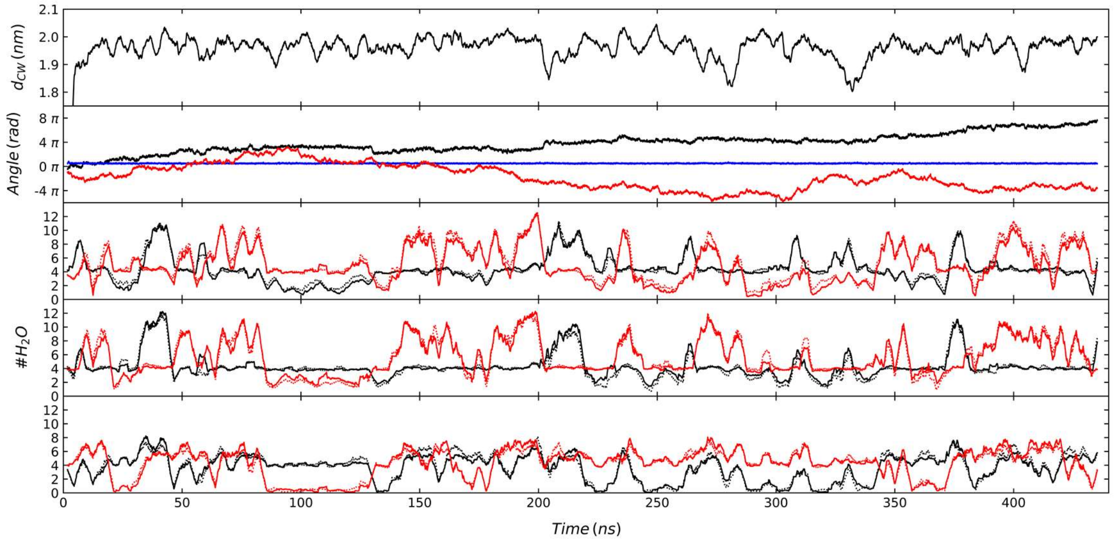

Figure S34.- Distance between the center of the O4 atoms of the two CDs composing the α-CD_2SDS_1 structure and the center of mass of the water molecules (first row). Yaw (red), pitch (blue) and roll (black) angles of the 2:1 complex as defined in Figure 2 with respect to the water/air interface (second row). Number of water molecules at less than 3 Å of any atom of the CD that is closer (solid lines) and further (dashed lines) to the SDS head in the complex (third to fifth rows). Rows 3, 4 and 5 represent GPUs {1,4}, {2,5} and {3,6} for one CD (black lines) respectively, and the corresponding GPUs of the opposite CD (red lines). This plot corresponds to the simulation of the **α-CD_2SDS_1 structure at the water/air interface** for a trajectory generated using the **HMR_2 method.**

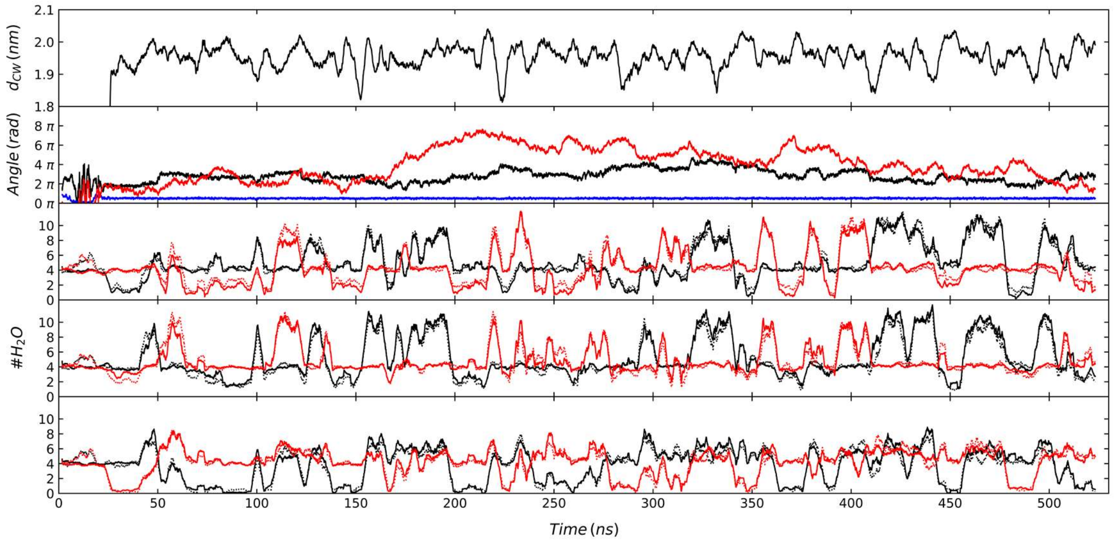

Figure S35.- Distance between the center of the O4 atoms of the two CDs composing the α-CD_2SDS_1 structure and the center of mass of the water molecules (first row). Yaw (red), pitch (blue) and roll (black) angles of the 2:1 complex as defined in Figure 2 with respect to the water/air interface (second row). Number of water molecules at less than 3 Å of any atom of the CD that is closer (solid lines) and further (dashed lines) to the SDS head in the complex (third to fifth rows). Rows 3, 4 and 5 represent GPUs {1,4}, {2,5} and {3,6} for one CD (black lines) respectively, and the corresponding GPUs of the opposite CD (red lines). This plot corresponds to the simulation of the **α-CD_2SDS_1 structure at the water/air interface** for a trajectory generated using the **HMR_7 method.**

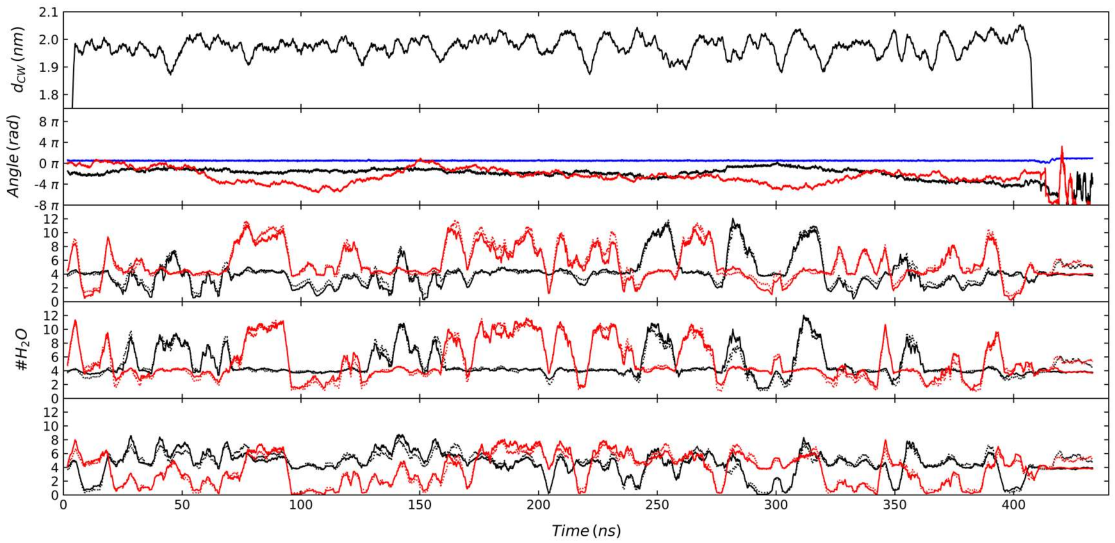

Figure S36.- Distance between the center of the O4 atoms of the two CDs composing the α-CD_2SDS_1 structure and the center of mass of the water molecules (first row). Yaw (red), pitch (blue) and roll (black) angles of the 2:1 complex as defined in Figure 2 with respect to the water/air interface (second row). Number of water molecules at less than 3 Å of any atom of the CD that is closer (solid lines) and further (dashed lines) to the SDS head in the complex (third to fifth rows). Rows 3, 4 and 5 represent GPUs {1,4}, {2,5} and {3,6} for one CD (black lines) respectively, and the corresponding GPUs of the opposite CD (red lines). This plot corresponds to the simulation of the **α-CD_2SDS_1 structure at the water/air interface** for a trajectory generated using the **H2Q_2 method.**

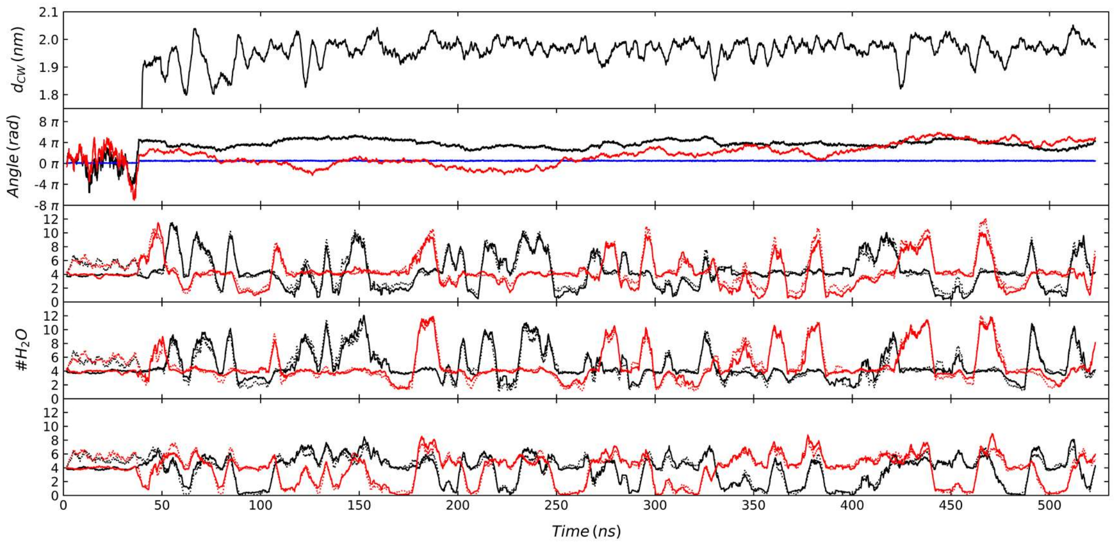

Figure S37.- Distance between the center of the O4 atoms of the two CDs composing the α-CD_2SDS_1 structure and the center of mass of the water molecules (first row). Yaw (red), pitch (blue) and roll (black) angles of the 2:1 complex as defined in Figure 2 with respect to the water/air interface (second row). Number of water molecules at less than 3 Å of any atom of the CD that is closer (solid lines) and further (dashed lines) to the SDS head in the complex (third to fifth rows). Rows 3, 4 and 5 represent GPUs {1,4}, {2,5} and {3,6} for one CD (black lines) respectively, and the corresponding GPUs of the opposite CD (red lines). This plot corresponds to the simulation of the **α-CD_2SDS_1 structure at the water/air interface** for a trajectory generated using the **H2Q_7 method.**

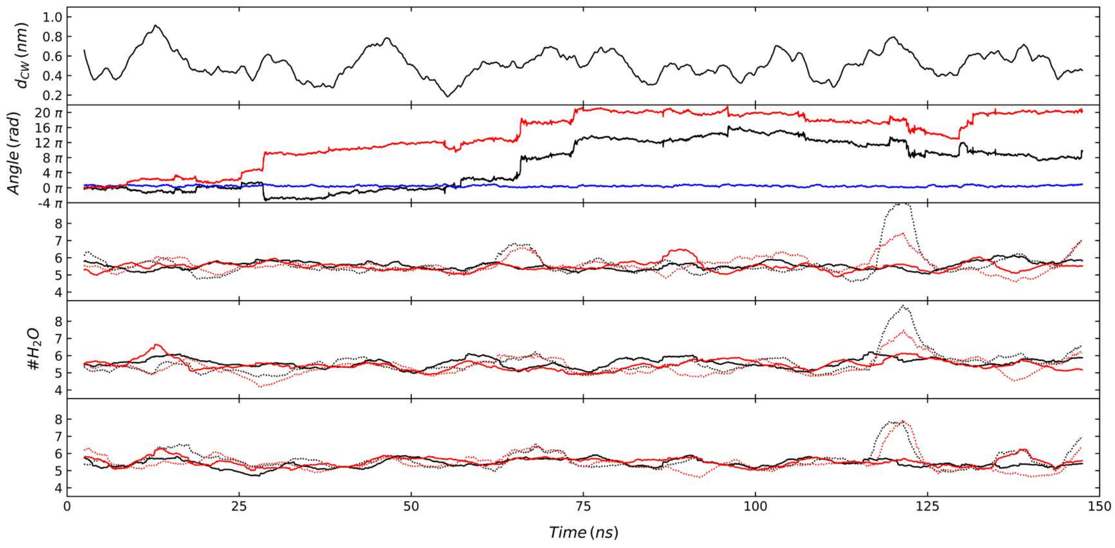

Figure S38.- Distance between the center of the O4 atoms of the two CDs composing the α-CD_2SDS_1 structure and the center of mass of the water molecules (first row). Yaw (red), pitch (blue) and roll (black) angles of the 2:1 complex as defined in Figure 2 with respect to the water/air interface (second row). Number of water molecules at less than 3 Å of any atom of the CD that is closer (solid lines) and further (dashed lines) to the SDS head in the complex (third to fifth rows). Rows 3, 4 and 5 represent GPUs {1,4}, {2,5} and {3,6} for one CD (black lines) respectively, and the corresponding GPUs of the opposite CD (red lines). This plot corresponds to the simulation of the **α-CD_2SDS_1 structure at the water/air interface** for a trajectory generated using the **AMBER BB-RESP method.**

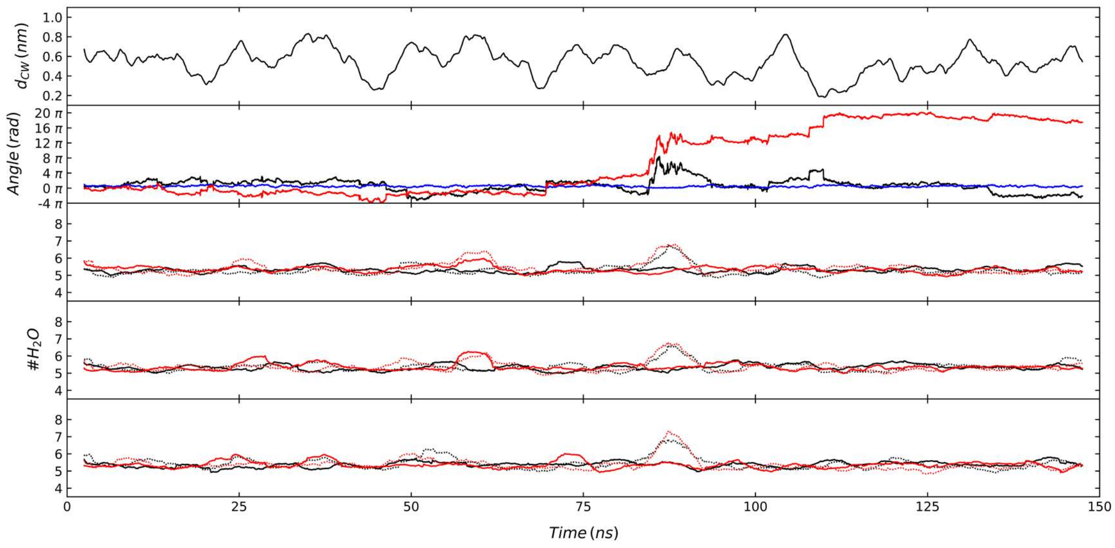

Figure S39.- Distance between the center of the O4 atoms of the two CDs composing the α-CD_2SDS_1 structure and the center of mass of the water molecules (first row). Yaw (red), pitch (blue) and roll (black) angles of the 2:1 complex as defined in Figure 2 with respect to the water/air interface (second row). Number of water molecules at less than 3 Å of any atom of the CD that is closer (solid lines) and further (dashed lines) to the SDS head in the complex (third to fifth rows). Rows 3, 4 and 5 represent GPUs {1,4}, {2,5} and {3,6} for one CD (black lines) respectively, and the corresponding GPUs of the opposite CD (red lines). This plot corresponds to the simulation of the **α-CD_2SDS_1 structure at the water/air interface** for a trajectory generated using the **AMBER BB-AM1 method.**
